# Using whole-genome SNP data to reconstruct a large multi-generation pedigree in apple germplasm

**DOI:** 10.1186/s12870-019-2171-6

**Published:** 2020-01-02

**Authors:** Hélène Muranty, Caroline Denancé, Laurence Feugey, Jean-Luc Crépin, Yves Barbier, Stefano Tartarini, Matthew Ordidge, Michela Troggio, Marc Lateur, Hilde Nybom, Frantisek Paprstein, François Laurens, Charles-Eric Durel

**Affiliations:** 1 IRHS, INRA, Agrocampus-Ouest, Université d’Angers, SFR 4207 QuaSaV, Beaucouzé, France; 2 Les Croqueurs de Pommes du Confluent Ain-Isère-Savoie, Les Avenières, France; 3 Department of Agricultural and Food Sciences, University of Bologna, Bologna, Italy; 4 School of Agriculture, Policy and Development, University of Reading, Whiteknights, Reading, UK; 5 Fondazione Edmund Mach, San Michele all’Adige, Trento, Italy; 6 CRA-W, Centre Wallon de Recherches Agronomiques, Plant Breeding & Biodiversity, Gembloux, Belgium; 7 Department of Plant Breeding, Swedish University of Agricultural Sciences, Balsgård, Kristianstad, Sweden; 8 RBIPH, Research and Breeding Institute of Pomology Holovousy Ltd., Horice, Czech Republic

**Keywords:** *Malus domestica*, Genotyping, Parentage analysis, Parent-offspring, Founders, Germplasm collection, Empirical selection, Modern breeding

## Abstract

**Background:**

Apple (*Malus* x *domestica* Borkh.) is one of the most important fruit tree crops of temperate areas, with great economic and cultural value. Apple cultivars can be maintained for centuries in plant collections through grafting, and some are thought to date as far back as Roman times. Molecular markers provide a means to reconstruct pedigrees and thus shed light on the recent history of migration and trade of biological materials. The objective of the present study was to identify relationships within a set of over 1400 mostly old apple cultivars using whole-genome SNP data (~ 253 K SNPs) in order to reconstruct pedigrees.

**Results:**

Using simple exclusion tests, based on counting the number of Mendelian errors, more than one thousand parent-offspring relations and 295 complete parent-offspring families were identified. Additionally, a grandparent couple was identified for the missing parental side of 26 parent-offspring pairings. Among the 407 parent-offspring relations without a second identified parent, 327 could be oriented because one of the individuals was an offspring in a complete family or by using historical data on parentage or date of recording. Parents of emblematic cultivars such as ‘Ribston Pippin’, ‘White Transparent’ and ‘Braeburn’ were identified. The overall pedigree combining all the identified relationships encompassed seven generations and revealed a major impact of two Renaissance cultivars of French and English origin, namely ‘Reinette Franche’ and ‘Margil’, and one North-Eastern Europe cultivar from the 1700s, ‘Alexander’. On the contrary, several older cultivars, from the Middle Ages or the Roman times, had no, or only single, identifiable offspring in the set of studied accessions. Frequent crosses between cultivars originating from different European regions were identified, especially from the nineteenth century onwards.

**Conclusions:**

The availability of over 1400 apple genotypes, previously filtered for genetic uniqueness and providing a broad representation of European germplasm, has been instrumental for the success of this large pedigree reconstruction. It enlightens the history of empirical selection and recent breeding of apple cultivars in Europe and provides insights to speed-up future breeding and selection.

## Background

Information about pedigrees is strategic for a wide range of uses, from animal and plant breeding to the study of human or wildlife genetics. In breeding, pedigree knowledge is essential for estimating heritabilities and genetic correlations of economically interesting traits [1]. Whilst markers alone can provide precise estimates of some genetic parameters [2], pedigree information makes it possible to also account for background similarity due to shared parents [3].

In wildlife genetics, parameters for ecologically relevant traits, most often related to fitness, can be estimated when the pedigree is either known or inferred. In conservation genetics, pedigree information enables estimation of relatedness between individuals and can assist genetic management programs [4, 5]. In addition, pedigrees can inform about mating behavior and variation in reproductive success in wild populations. Furthermore, pedigree knowledge is useful to trace history of migrations or exchanges at very recent time scales, not only for humans but also for the biological material they convey. Finally, pedigree knowledge and accuracy is instrumental to assemble balanced sets representing important breeding parents with the purpose of detecting and validating QTLs using the Pedigree Based Analysis approach [6–8].

Molecular markers can be used to reconstruct pedigrees in populations when matings and/or parent-offspring relationships are unobservable, such as for aquatic animals [9], and also to investigate recorded pedigrees where parentage may be uncertain [10]. Conversely, using known pedigrees as a basis to check for consistency of Mendelian inheritance can assist in quality control when marker data are obtained in very large quantity at the same time [11]. Subsequently, pedigrees can provide crucial information for imputation of missing marker data, particularly for the most recent generation(s) [12].

Attempts to test or reconstruct pedigrees using molecular markers began with isozyme analysis in the 1980s [13, 14], continued with minisatellite fingerprinting [15–17] and then microsatellite (SSR) markers [18, 19]. More recently, single nucleotide polymorphisms (SNPs) have provided an unprecedented ability to reconstruct pedigrees due to their high abundance, codominant mode of inheritance and the low cost of genotyping per locus through high-throughput genotyping techniques [10, 20–22]. Six categories of method for parentage analysis were reviewed by Jones et al. [23] and all were felt to have value. However, as the authors also highlighted, a successful study does not depend solely on the method used for data analysis, but also on the number and quality of the markers used and the adequate sampling of the population.

Apple (*Malus* x *domestica* Borkh.) is a very important fruit crop with an annual worldwide production of 83 million tons (FAOSTAT, 2017) across 4.9 million hectares, mainly in temperate regions. The cultivated apple is believed to derive from the Central Asian forest species *M. sieversii* (Ldb.) M. Roem, with later genetic contributions from the European crabapple species *M. sylvestris* Mill. [24]. The grafting process, probably developed 3800 years ago in Mesopotamia [25], enables clonal propagation of selected individuals which preserves their genetic combinations. Consequently, cultivars can be maintained in collections several centuries after their origination from seed.

Extremely old cultivars, such as ‘Pomme d’Api’ (synonyms ‘Api Rose’, ‘Lady Apple’) and ‘Court-Pendu’ (‘Capendu’) are said to be of Roman origin, although there is little tangible evidence for this [26, 27]. The Roman naturalist Pline described 24 apple cultivars in his encyclopedia *Historia naturalis* (cited by Leroy [26]). Mythical cultivars such as ‘Costard’ and ‘Old English Pearmain’ were mentioned as far back as the thirteenth century [28, 29], but seem not to have been conserved under these names. Later on, during the 16th and 17th centuries, some cultivar names became more established, such as ‘Api’ (with derivations: ‘Petit Api’, ‘Gros Api’, ‘Api Etoilée’), ‘Reinette Franche’, ‘Calville Blanc d’Hiver’, ‘Calville Rouge’, ‘Court-Pendu Gris’, ‘Golden Pippin’, ‘Rambour’, and ‘Petit-Bon’ (cited by Leroy [26]). From the same ages, apple fruits from old cultivars were also represented in paintings, e.g. the Bartolomeo Bimbi paintings at Villa Medicea di Poggio a Caiano, Prato, Italy.

Another consequence of the grafting process is that selected individuals are easily propagated and distributed in large numbers. Present-day apple production and breeding are therefore dominated by a relatively small number of widely distributed cultivars, potentially leading to a reduction in genetic diversity [30, 31]. Fortunately, numerous germplasm collections have been established for assessing the material grown in a region or a country, and/or for preserving this variability for future generations. As a result, thousands of apple cultivars are held in collections worldwide [32–34].

Many germplasm collections have been analyzed with molecular markers in order to identify redundancy and to determine genetic diversity and structure. However, most studies have dealt with material from limited geographic areas [35–47]. In addition, the use of different marker techniques, or different sets of markers, has prevented analysis at a global level. Only recently, attempts were made to broaden the scope of genetic diversity studies by using a common set of SSR markers [32, 48].

Parentage analysis was performed in some of the above-mentioned studies but an inadequate number of markers has limited the ability to infer parentages, particularly between genotypes with widely occurring alleles. As a consequence, key individuals with a high number of offspring over multiple generations have been difficult to identify [32]. Other attempts to test or recover parentage of emblematic cultivars have been published [20, 38, 39, 49–53] but these have often been based on a limited set of germplasm. Recently, additional pedigrees have been proposed after genotyping the large international germplasm collection of the UK National Fruit Collection (over 2000 accessions) with Diversity Array Technology (DArT) markers [54], but the dominant nature of these markers, and the level of admixture in the population limited the inferences that could be made to those involving potential inbreeding or mixed ploidy relations.

Pedigree reconstruction of apple cultivars can shed light over historical transactions, e.g., the exchange of grafts at a local scale between farmers, or at a broader scale between castles and monasteries within and across countries. It can also shed light on the movement of apples overseas, the seeds of which have given rise to numerous so-called “chance seedlings” [30].

The present study aims to investigate parentages and reconstruct pedigrees within a very large set of apple cultivars using the Axiom**®**Apple480K array [55]. The high number of SNPs enabled us to use simple exclusion tests based on Mendelian error counts to identify numerous parent-offspring relations and consequently reconstruct multi-generation pedigrees involving cultivars selected during several centuries. To our knowledge, this is the largest analysis of its kind ever performed in a perennial fruit species.

## Results

### Parent-offspring duos

**Fig. 1:**
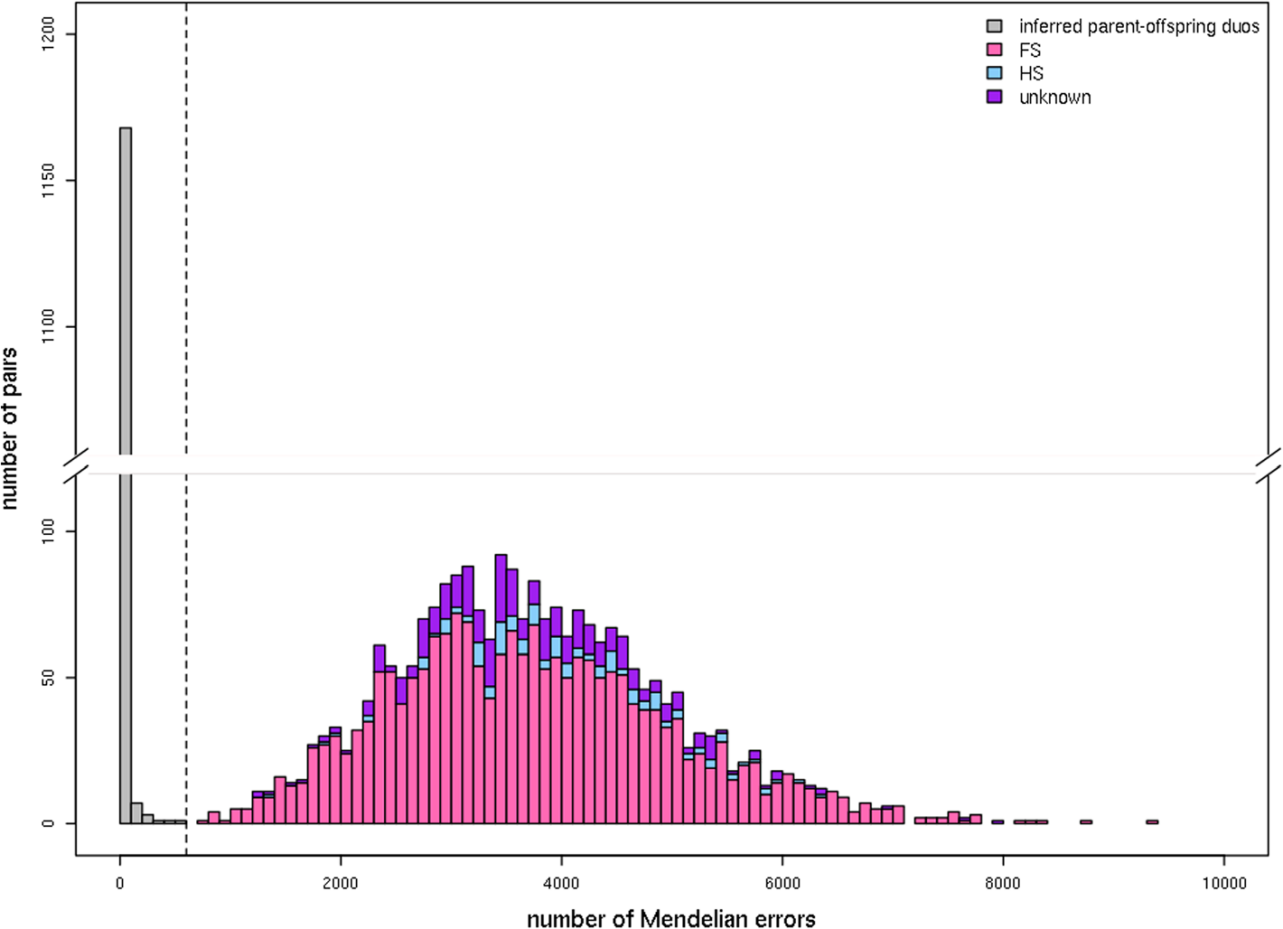
Distribution of Mendelian Error (ME) counts in 3655 pairs of diploid *Malus domestica* individuals tested as parent-offspring duos. The inferred parent-offspring pairings are accounted for in the gray bars, the full-sib pairings in the pink bars, the half-sib pairings in the light blue bars and the other pairings in the purple bars

A total of 1425 diploid individuals (Additional file 1: Table S1) was analyzed with the Axiom**®**Apple480K array [55]. A set of 253 K SNPs (Additional file 2: Table S2) was used to compute Identity By Descent (IBD) sharing probabilities for all possible pairings of diploid individuals, using the ‘PI_HAT’ parameter of PLINK [56]. For the 3655 pairings with a PI_HAT value greater than 0.4, the distribution of Mendelian errors (ME) ranged from 0 to 9376 with a distinct gap around 600 (Fig. 1). Below this gap was a total of 1181 pairings deemed to be parent-offspring relations (duos), of which 184 pairings were from two small segregating populations, each containing 46 progenies and their parents (i.e. one for each of the two known parents with each of the 92 offspring). The number of ME varied between 767 and 1240 for 23 pairings of presumed full-sib individuals, either from the two segregating populations (20 pairs) or deduced retrospectively from further complete parent-offspring trio analysis (3 pairs). Other pairings of full-sib individuals from the two segregating populations presented higher ME counts. Four parent-offspring duos reported in the literature had a ME count above the threshold of 600: ‘Westland’ and ‘Heyer12’, with 1241 ME; F2–26829–2-2 and PRI14–126, with 1549 ME; ‘Rescue’ and ‘Norland’, with 1816 ME; and ‘Rescue’ and ‘Parkland’, with 2968 ME.

### Network of relationships involving duos

**Fig. 2:**
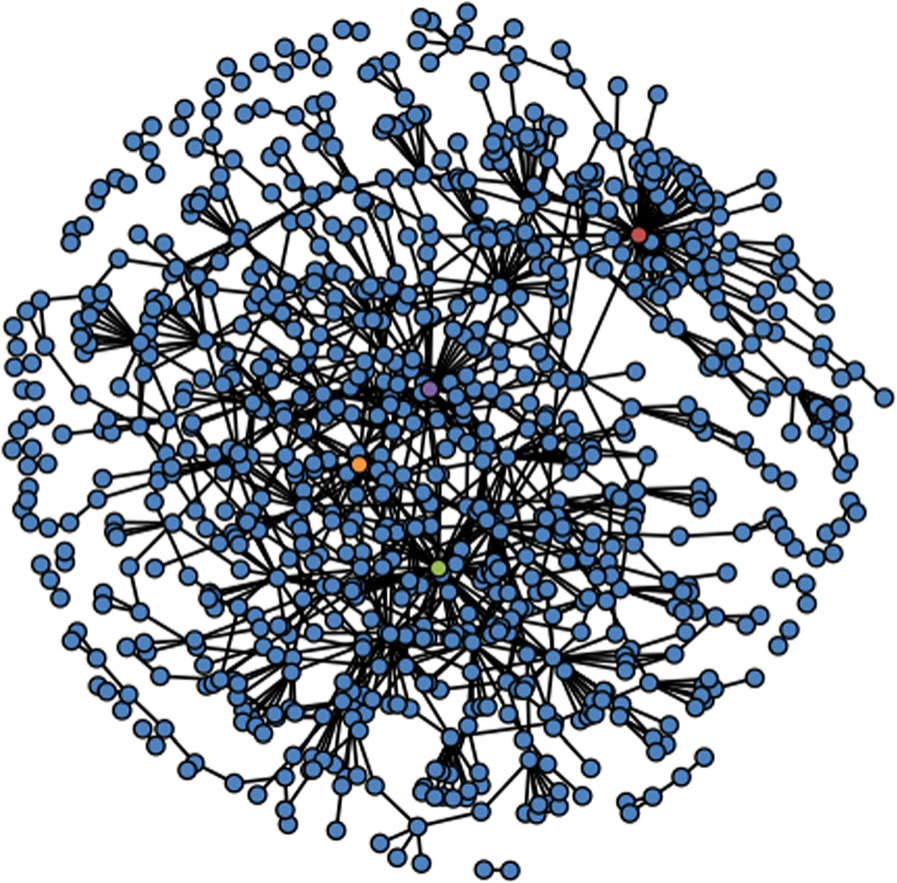
Network of pedigree relationships of 832 diploid *Malus domestica* individuals. Each individual is represented by a dot while connecting lines in the network represent first-degree relationships. All individuals are represented by blue dots, except for ‘Reinette Franche’, red dot, ‘Cox’s Orange Pippin’, green dot, ‘Alexander’, purple dot, and ‘Borowitsky’, orange dot

A total of 924 individuals, among the 1425 diploids examined were involved in pairings with a ME count below 600 and these were considered to be involved in a parent-offspring duo; among these were all 92 offspring from the two segregating populations. After excluding the latter, we found 397 (30%) individuals involved in only one parent-offspring duo, 307 (23%) involved in two duos, 73 (5.5%) involved in three to five duos, 31 (2.3%) involved in six to ten duos and 24 (1.8%) involved in more than ten duos, with a maximal count of 66 parent-offspring duos involving the old French cultivar ‘Reinette Franche’ (assigned the unique genotype code ‘MUNQ 278’; see Additional file 1: Table S1 and Methods, Plant Material). The remaining 501 (38%) diploid individuals were not involved in any pairings. A network obtained from all the proposed parent-offspring duos, as illustrated in Fig. 2, demonstrates the high level of connectivity within our sample, including a large set containing 766 (58%) individuals, six small sets containing three to five individuals, and 23 sets containing only two individuals.

### Complete parent-offspring trios

**Fig. 3:**
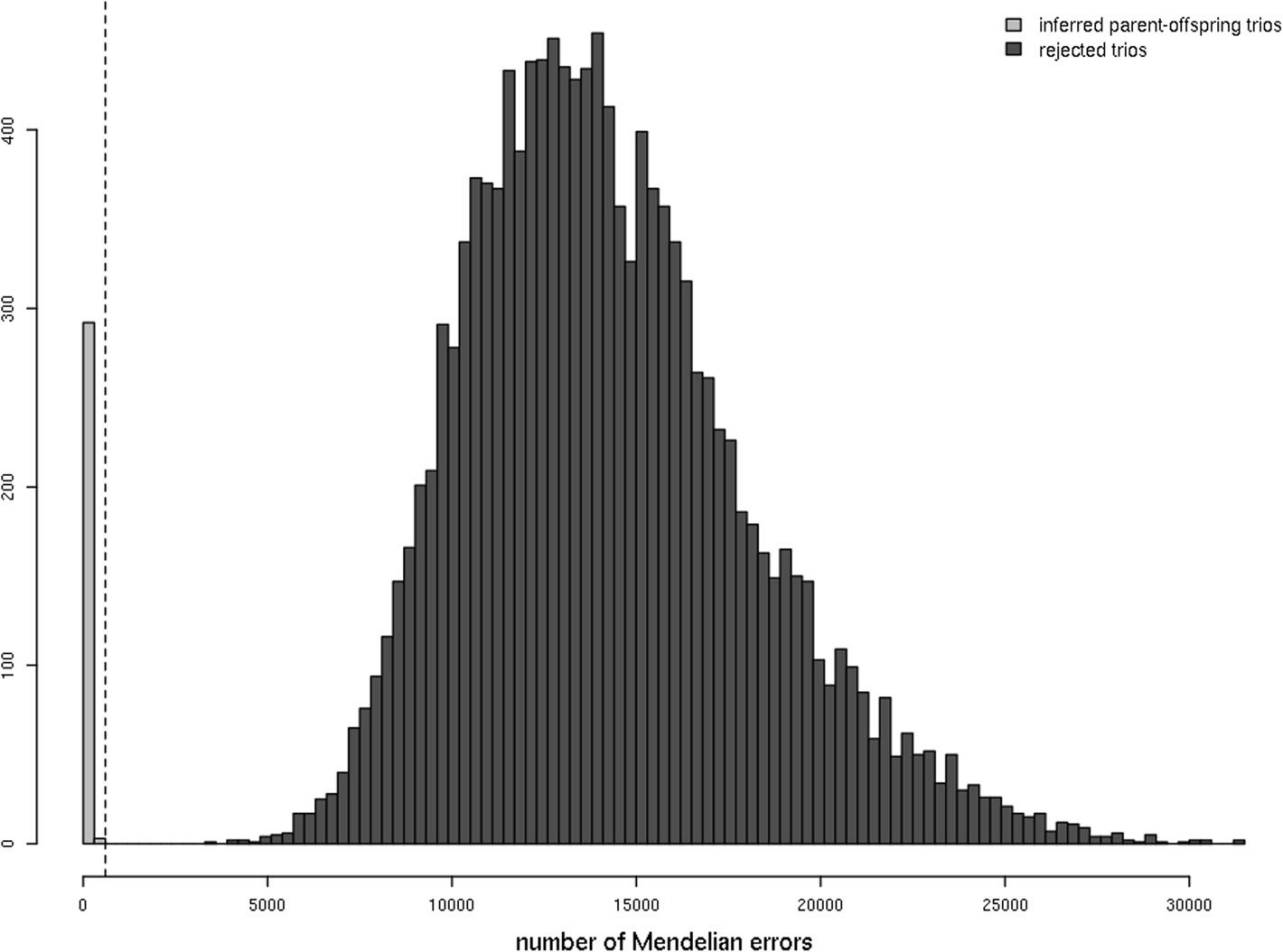
Distribution of Mendelian Error (ME) counts in 13,603 trios of diploid *Malus domestica* individuals tested as complete parent-offspring trios. The inferred parent-offspring trios are accounted for in light gray bars, and the rejected trios in the dark gray bars

A total of 13,603 potential trios (one individual and its two potential parents) involving diploid individuals were tested and resulted in counts from 0 to 31,398 ME. The distribution of ME in all tested trios (Fig. 3) contained a large gap between 575 and 3448, and the first three trios with a count of ME greater than or equal to 3448 all suggested the same individual (‘Cox’s Orange Pippin’) as the offspring and thus were not all credible. A total of 295 trios had a ME count below 600 and these were inferred as complete parent-offspring sets. Among these, 32 inferred parent couples shared between two and five offspring, resulting in small full-sib families, while 212 inferred parent couples had only one offspring in the dataset. The largest inferred full-sib family included as the parents ‘Jonathan’ and ‘Cox’s Orange Pippin’ and comprised five offspring, namely ‘President Boudewijn’, ‘Prinses Beatrix’, ‘Prinses Irene’, ‘Prinses Marijke’ and ‘Céres’, the first four being documented to have been raised from these two parents in 1935 by ‘Instituut voor de Veredeling van Tuinbouwgewassen’ (IVT) in The Netherlands (28; Additional file 3: Table S3). ‘Jonathan’ and ‘Cox’s Orange Pippin’ were, independently, also the genotypes inferred as parents in the highest number of trios, with 24 and 45 offspring respectively. One hundred additional genotypes were inferred as parent in two or more trios, while 73 were inferred as parent in only one trio.

All trios scoring fewer than 600 ME are presented in Additional file 3: Table S3. We were able to find prior historical documentation (sources mentioned in Additional file 4) regarding both parents for 119 of the trios and our findings were in agreement with 79 of them. As an example, the documented parents of several famous cultivars such as ‘Gala’ (= ‘Kidd’s Orange Red’ x ‘Golden Delicious’), ‘Discovery’ (= ‘Worcester Pearmain’ x ‘Beauty of Bath’), ‘Fiesta’ (= ‘Cox’s Orange Pippin’ x ‘Idared’), ‘Fuji’ (= ‘Ralls Janet’ x ‘Delicious’) and ‘Akane’ (= ‘Jonathan’ x ‘Worcester Pearmain’) were all in agreement with our findings. For four trios, the documented name of at least one parent was not identical to the preferred name we chose for the individual identified, but was similar, and this is a complication broadly accepted within the international genetic resource and horticultural community. For example, ‘Zhigulevskoe’ is documented as ‘Duchess of Oldenburg’ x ‘Wagenar Prizovoe’ (Additional file 3: Table S3) and our results identified ‘Wagener’ x ‘Borowitsky’ as the potential parentage; ‘Borowitsky’ is a synonym of ‘Duchess of Oldenburg’ and we consider ‘Wagenar Prizovoe’ (meaning Wagner’s prize-winning in Russian) is most likely to refer to ‘Wagener’. For 15 trios out of the 119, only one documented parent was in agreement with our findings, while for 11 trios, neither of the documented parents agreed with our results. Finally, for 10 trios, different sources indicated different parents, and our findings were in agreement with only one of the available sources.

We could only find documentation of one of the two parents for 23 of the identified trios (Additional file 3: Table S3). The documented parent agreed with one of the identified parents for 12 of these; for three additional trios, the documented parent had a name similar to that of one identified parent. However, for eight trios, the documented parent was not among the identified parents.

We also checked for chronological consistency within the trios. We found documented dates for both inferred parents and offspring for 192 trios (Additional file 3: Table S3). The inferred offspring appeared younger than its proposed parents in 180 cases. We found documented dates for only one of the inferred parents for 7 other trios and the inferred offspring was younger than the inferred parent in all cases. In most of the twelve cases where the dates appeared to be potentially inconsistent, the documented dates were dates of first recording or of reception in collection which can be rather far from the true date of origination and thus do not enable assessment of chronological consistency.

### Oriented parent-offspring duos

After removing parent-offspring duos that were subsequently identified within trios and those involved in the segregating populations, a total of 407 inferred parent-offspring duos remained. For 71 of these, one of the two individuals in the pair had already been proposed as a potential offspring in a trio and thus was inferred as the potential parent in the remaining duo(s). For six additional duos, one of the individuals was identified as an offspring in these newly-oriented duos and thus inferred to be the parent in the remaining duo(s). All 77 of these parent-offspring duos are presented in Additional file 5: Table S4. The remaining 330 duos could not be oriented at this stage.

Overall, 22 genotypes were inferred to be parents in the oriented duos, with 12 of them being inferred as parents in two or more oriented duos and thus suggesting half-sib relationships among the corresponding offspring. Interestingly, two genotypes were inferred as parents in more than ten oriented duos, namely ‘Reinette de Hollande’ and ‘Calville Rouge d’Hiver’, with 19 and 11 inferred offspring respectively.

The 330 non-oriented duos involved 415 genotypes, 75 of which were inferred to be involved in two or more duos. Notably, the three genotypes ‘Reinette Franche’, ‘White Astrachan’ and ‘Saint Germain’ were inferred to be involved in 57, 12 and 10 parent-offspring relationships respectively, as well as having been inferred as parents in nine, four and six trios.

We found documentation regarding one or both parents of 25 of the individuals inferred as offspring in the oriented duos and our findings agreed with one of the parents for 15 of these (Additional file 5: Table S4). The other parent was either absent from our dataset (for ‘Pitchounette’ and for ‘Delrouval’) or deemed to be false (e.g., ‘Bismarck’ for ‘S.T. Wright’). We found documented dates for both the inferred offspring and parent of 49 oriented duos and 38 of these were chronologically consistent. Again, most exceptions involved dates of first recording or of reception in collections which can be rather far from the true date of origination.

All 330 non-oriented duos are presented in Additional file 6: Table S5. We found documentation of at least one parent for one of the individuals in 82 of these duos and this relationship was in agreement with our findings in 44 cases, allowing us to orient the pair. We found documented dates for both members of 218 non-oriented duos and for 202 of them, one member was clearly more recent than the other and thus regarded as the probable offspring. For 41 of these, the dates supported the orientation already deduced from the documented parentage. For one duo, oriented according to documented parentage, the documented dates were however contradictory, namely for ‘Fenouillet Rouge’ inferred as an offspring of ‘Opetian’. For 161 duos, documented dates were the only basis on which to orient the duo. Two individuals, namely ‘Míšeň jaroměřská červená’ and ‘Pine Apple Russet’, were each inferred as offspring in two duos based on dates, although this was in contradiction with the fact that the two proposed parents and the related individual did not form a trio.

In 15 cases, the documented dates were too imprecise or too close to determine orientation, including two duos involving ‘Opetian’, with ‘Pacheroux’ and ‘Pomme de Sore’, and two duos involving ‘Reinette Franche’, with ‘Calville Malingre’ and ‘Nonpareil’. Another 45 duos were oriented because one of the individuals was the offspring in a duo oriented thanks to previously documented parentage or date. However, dates contradicted the orientation for two of these, ‘Golden Dorsett’ as an offspring of ‘Anna’ and ‘Belle Fille de l’Indre’ as an offspring of ‘Franc Roseau du Valais’. Finally, the 80 remaining duos could not be oriented.

### Grandparents-parent-offspring groups

**Fig. 4:**
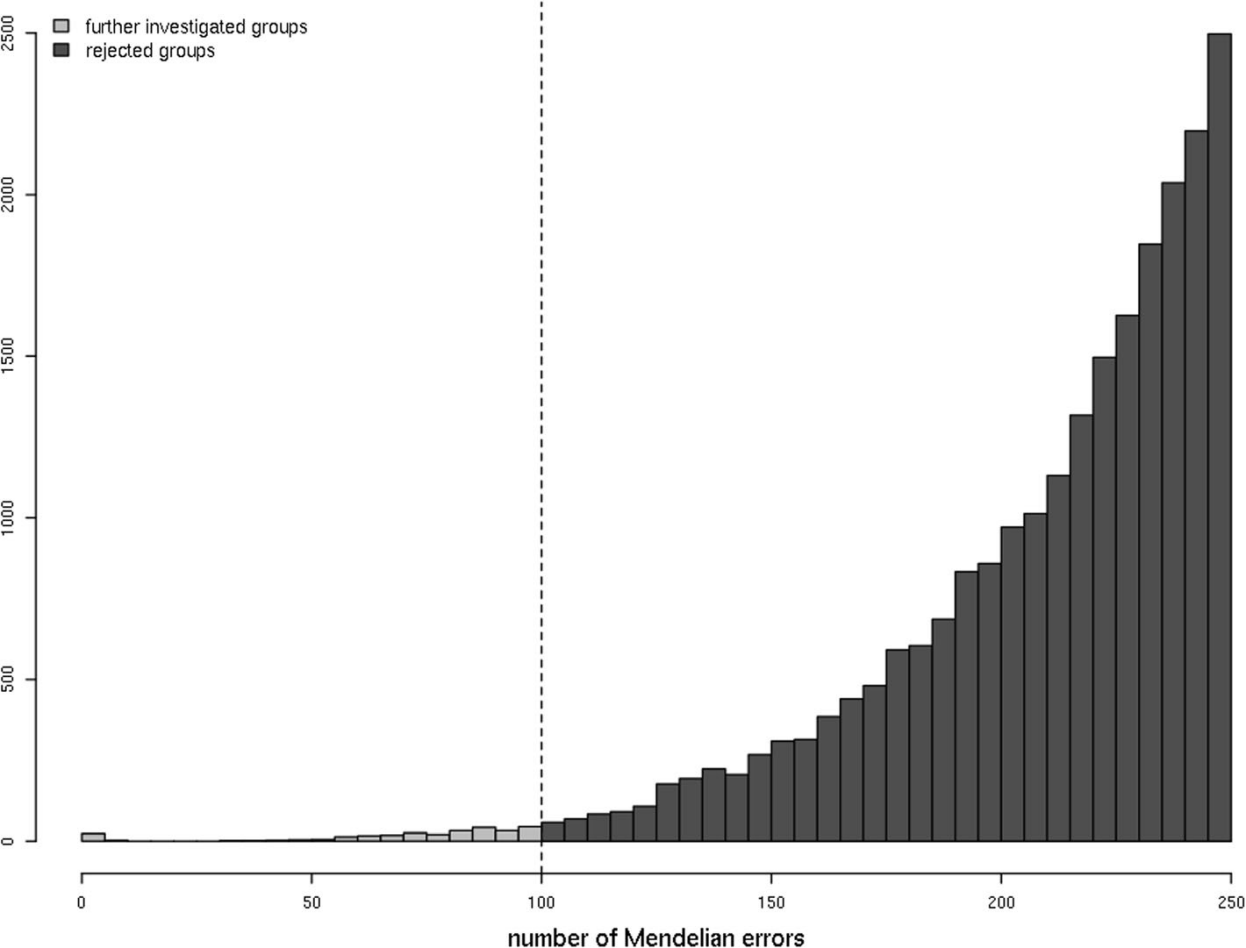
Distribution of Mendelian Error (ME) counts in 1,823,127 groups of diploid *Malus domestica* individuals tested as grandparents-parent-offspring groups with 25,310 SNPs. The further investigated groups are accounted for in the light gray bars, and the rejected groups in the dark gray bars

**Fig. 5:**
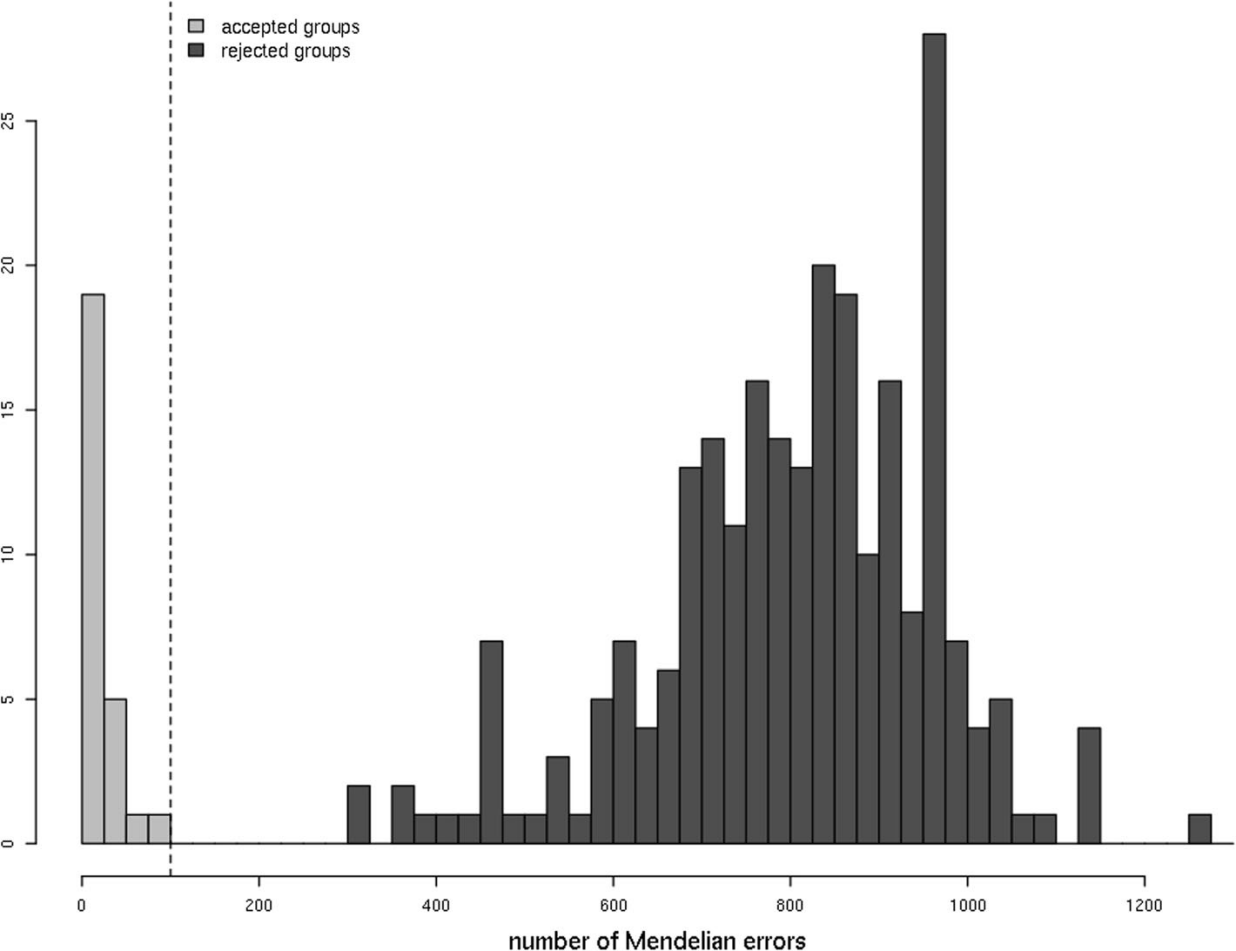
Distribution of Mendelian Error (ME) counts in 273 groups of diploid *Malus domestica* individuals tested as grandparents-parent-offspring groups with 253,095 SNPs. The accepted groups are accounted for in the light gray bars, and the rejected groups in the dark gray bars

Using a first subset of 25 K SNPs, the distribution of ME in all tested groups with a potential grandparent couple, parent and offspring, showed a distinct gap between 10 and 35 (Fig. 4). Following initial liberal selection with ME fewer than or equal to 100, the distribution of ME using the 253 K SNPs set in the tested subset of potential grandparents-parent-offspring groups contained a gap between 85 and 312 (Fig. 5). The first eight groups with a ME count greater than or equal to 312 all suggested the same genotype as the offspring (‘Cox’s Orange Pippin’) and thus again, were not all credible. A total of 26 groups had fewer than 100 ME and were considered further in pedigree analysis (Additional file 7: Table S6). Two potential grandparent couples had more than one inferred grandchild while 19 had only a single inferred grandchild. Moreover, four of these potential grandparent couples also had one or two inferred offspring in the dataset (i.e. were identified as a parent couple in inferred trios). A total of 36 genotypes were inferred at least once as grandparents, and eight of them were included in two to five groups. For six of the predicted groups, orientation of the potential parent-offspring was already resolved since the parent was also inferred as an offspring in a trio. Identification of ‘Orleans’ as the potential offspring in an inferred grandparents-parent-offspring group supported the prior orientation for a duo where ‘Anna’ was considered as the offspring and ‘Orleans’ as the parent on the basis of documented dates (Additional file 6: Table S5).

All inferred grandparents-parent-offspring groups are presented in Additional file 7: Table S6. Documentation of parentage was found for 15 cultivars inferred as offspring in these groups, and six of these were in agreement with our results. We found documented dates for 22 individuals inferred as offspring in the potential grandparents-parent-offspring groups, and the dates were consistent with the proposed orientation. Interestingly, five inferred offspring derived from the same potential grandparent couple, namely ‘Keswick Codlin’ x ‘Hawthornden’.

### Pedigree deduced from all results

All of the inferred trios, oriented duos and groups of grandparents-parent-offspring were used to produce a large pedigree which comprised 775 genotypes, including 133 founders, 295 genotypes with two inferred parents, 295 genotypes with only one inferred parent, 26 genotypes with one inferred parent, one missing parent and inferred grandparents and the corresponding 26 missing parents from the grandparents-parent-offspring groups. Since the non-oriented duos were not included in this pedigree, some individuals considered here as founders are additionally related, e.g., ‘Reinette Franche’ and ‘Nonpareil’.

**Fig. 6:**
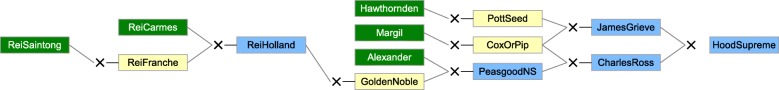
Reconstructed pedigree of ‘Hood’s Supreme’. Cultivar names are in their short version (see Additional file 1: Table S1). The coloring of the name bars indicates the level of information known for the individual(s) in the pedigree: blue, individuals with both parents known, cream, individuals with one parent known, dark green, founders. Relationships are represented by black lines as the mother and the father cannot be identified with our data. The figure was drawn using data extracted from Additional file 8 and the Pedimap software [57]

The deduced pedigree encompassed seven generations (Fig. 6; Additional file 8; Additional file 9: Fig S1), with the two founders ‘Reinette de Saintonge’ and ‘Grimes Golden’ as the oldest ancestors, and consequently part of the first generation. These two cultivars were then inferred to have 244 and 38 offspring, respectively, within the second to seventh generation of the pedigree. All inferred offspring of ‘Reinette de Saintonge’ were derived through ‘Reinette Franche’, and a large part of these (77 out of 244) through ‘Reinette de Hollande’ resulting from the cross between ‘Reinette Franche’ and ‘Reinette des Carmes’. In contrast, the two predicted full-sibs of ‘Reinette de Hollande’ (‘Mabbott’s Pearmain’ and ‘Adams’s Pearmain’) did not have any predicted offspring in the dataset explored. The founder ‘Alexander’ had inferred descendants over four generations, with 105 offspring predicted in total. Another founder with numerous inferred descendants (87) over four generations was ‘Margil’, mostly through ‘Cox’s Orange Pippin’ inferred as one of its first generation offspring. Conversely, ‘Black Gilliflower’ had only four inferred descendants over four generations, i.e. one per generation.

### Deduced inbreeding

Considering the deduced pedigree, 7 genotypes were found to be inbred due to a shared ancestor for their two inferred parents, namely ‘George Carpenter’ (through ‘Reinette de Hollande’), ‘Alro’, ‘Petit Pippin’ and ‘F_Democrat’ (through ‘Reinette Franche’), ‘Fairie Queen’ and ‘Hood’s Supreme’ (through ‘Cox’s Orange Pippin’), and ‘F_Rosavril’ (through ‘Winesap’). Considering the 295 complete parent-offspring trios and the 26 grandparents-parent-offspring groups, the frequency of pedigree-based inbred genotypes was thus 2.2%. When comparing this inbreeding assessment to heterozygosity in all diploid genotypes, we found that heterozygosity varied between 0.24 and 0.397, and only three individuals could be considered as statistically-significant outliers (*P* < 0.05) as compared to the distribution of heterozygosity in all genotypes (see Additional file 10: Figure S2). These three outlier genotypes were ‘Maiolino (PA)’, ‘Hashabi (MH. 10–1)’ and ‘Fairie Queen’, with heterozygosities of 0.240, 0.244 and 0.248, respectively.

### Triploids

Eight triploid cultivars were genotyped with the Axiom**®**Apple480K array and analyzed as if they were diploid (see Methods). The distribution of ME in all tested pairs of triploid-diploid individuals contained a distinct gap around 300 (Additional file 11: Figure S3). Using this as a threshold led to a total of 66 potential triploid offspring-diploid parent pairs. For all 66 of these pairs, the number of tri-hom/di-het SNPs (homozygous in the triploid offspring and heterozygous in the diploid parent SNPs) varied between 593 and 38,461, with a gap around 1200. Five inferred triploid offspring-diploid parent pairs had a count of tri-hom/di-het SNPs below 1200, and this was taken as a threshold to indicate a potential parent that could have contributed a diploid gamete.

A total of 515 potential trios involving a triploid as the offspring and two diploid individuals as parents were tested and resulted in counts from 136 to 29,574 ME. The distribution of ME in these tested trios had a large gap between 246 and 5170. Two inferred trios had a count of ME below 300, namely ‘Jonagold’ = ‘Golden Delicious’ x ‘Jonathan’ and ‘Ribston Pippin’ = ‘Margil’ x ‘Nonsuch Park’. The first inferred trio was already well documented since the offspring was derived through modern plant breeding, while the second was only in part proposed by Ordidge et al. [54] who recently inferred the duo between ‘Margil’ and ‘Ribston Pippin’.

Out of the 61 diploid individuals that had a low ME count for a potential parent-offspring relationship with a triploid, but a large number of tri-hom/di-het SNPs with this triploid, 56 also had an inferred parent-offspring relationship with the 2*n*-gamete parent of the triploid, when one was identified. Among them, ‘Cox’s Orange Pippin’ was inferred as an offspring of ‘Margil’ (Additional file 6: Table S5), the apparent 2*n*-gamete parent of ‘Ribston Pippin’, on the basis of documented date. For all these 56 diploid individuals, we also checked that they almost never received an allele from the haploid gamete parent when the 2*n*-gamete parent of the triploid was homozygous and the triploid heterozygous. The maximum count of SNP indicating a putative allele transmission from the haploid gamete parent was 18. As a consequence, we could exclude the triploid as a parent of these 56 diploid individuals and consider confidently the parent-offspring relationship they had with the inferred 2*n*-gamete parent. For three inferred triploid-diploid pairings with a low number of ME and a large number of tri-hom/di-het SNPs, we were not able to identify a 2*n*-gamete parent and consequently we propose the diploid individuals as potential parents contributing a haploid gamete to the triploids. However, they could alternatively be in a parent-offspring relationship with the unknown 2*n*-gamete parent making them sibs of the triploid. The two remaining inferred triploid-diploid pairings with a low number of ME and a large number of tri-hom/di-het SNPs involved, as diploids, the individuals contributing a haploid gamete in the two inferred trios. These results are presented in Additional file 12: Table S7.

## Discussion

In the present study, we utilized the dense SNP dataset available from the Axiom**®**Apple480K array to investigate parentage and reconstruct pedigrees in a large set of apple cultivars. We used a simple exclusion test based on counting Mendelian Errors (ME) in putative relationships. Complete parent-offspring trios as well as individual parent-offspring duos were investigated. Where only a single parent could be proposed, we investigated whether the parents of the missing parent could be identified through an additional grandparent-offspring relationship.

### Methodology for detecting duos and trios

Given the high number of SNPs available and the wide range of Minor Allele Frequencies (MAF) covered by the markers placed on the array [55], we considered that a simple exclusion test based on a count of ME was robust enough to support our findings, and this allowed us to avoid more mathematically complex approaches which might have required error-free data and been compromised by the relationships intertwining across generations, as is the case in apple.

The error threshold of 600 ME out of 253,095 SNPs (0.24%) was chosen on the basis of identifying a distinct gap in the distribution of ME over the tested pairings. Theoretically, a complete absence of ME would be expected for any true parent-offspring relations, and this is potentially an indication of the remaining experimental error and/or biological variation.

Interestingly, the SNPs causing ME in the six duos below, but closest to the 600 ME threshold, were mostly concentrated on one or two chromosomes: for the duo ‘H 31–31’ – ‘Baujade’, 191 out of 220 ME occurred on chromosome 9; for three duos involving ‘Worcester Pearmain’ and for the duo ‘Sjögesta Pippin’ – ‘Grågylling’, more than 69% of the ME (ranging from 282 to 559) occurred on chromosome 13; while 130 and 227 of the 417 ME for the duo ‘HYB.N°29’ – ‘Prima’ were situated on chromosomes 1 and 5 respectively. This concentration of ME could potentially correspond to chromosomal fragments introduced from wild and/or related *Malus* species, where sequence divergence would be expected to produce a higher frequency of null or erroneous alleles. Above our threshold, we identified four pairings that were already documented as parent-offspring but had relatively high counts of ME (1241–2968): one of these was known to be derived from the wild species *Malus floribunda* (‘F2–26829–2-2’ and ‘PRI14–126’); the remainder comprised cultivars bred for frost resistance at Morden Research Station, Agriculture Canada, where there is a documented history of using wild species and ‘crab apples’ and all three have a parent, ‘Rescue’ or ‘Heyer12’, that has been classified as a species hybrid [58]. In all four cases, the high number of ME could derive from SNPs with null alleles. Overall, we considered the threshold of 600 ME as sufficiently robust and, whilst it may exclude some apples with a hybrid background, we expect the type I risk (acceptance of false parent-offspring) and type II risk (missing true parent-offspring) to otherwise be low.

For potential complete parent-offspring trios, the gap between groups below the threshold number of ME (< 600) and groups above the threshold was much larger than for the duos, suggesting that this parameter introduced a further level of discrimination. This is consistent with the requirement to allocate all alleles of the offspring to either parent in the trio. Again, genotyping errors and null alleles could be envisaged to explain the occurrence of ME in trios where complete absence of ME would have been expected.

Above the threshold, the 9 first trios (with ME ranging from 3448 to 5008) included 6 cases where ‘Cox’s Orange Pippin’ would have been the proposed offspring. Clearly, ‘Cox’s Orange Pippin’ could not be the product of 6 different parental pairings. However, five out of the 6 would have involved ‘Margil’ as the first parent and a documented or presently inferred offspring of ‘Cox’s Orange Pippin’ as the second parent (thus being grandparent-parent-offspring groups according to our interpretation); the sixth involved a pairing between two progenies of ‘Cox’s Orange Pippin’, one of which was derived from a back-cross with ‘Cox’s Orange Pippin’ as a recurrent parent. Indeed ‘Cox’s Orange Pippin’ was inferred here to be both the parent and (recurrent) grandparent of ‘Fairie Queen’ while ‘James Grieve’ was inferred to be its other parent (Additional file 3: Table S3). In the remaining three cases, 1-‘James Grieve’ was rejected (4242 ME) as a potential offspring from ‘Potts’ Seedling’ and ‘Fairie Queen’ whereas a more probable prediction (with only 28 ME) suggested that it is an offspring from ‘Potts’ Seedling’ and ‘Cox’s Orange Pippin’ in agreement with Ordidge et al. [54], 2- ‘Jonathan’ was rejected (4979 ME) as a potential offspring from ‘Montfort’ and ‘Florina’, both being either inferred or known (respectively) as offspring of ‘Jonathan’, and 3- ‘Reinette Franche’ was rejected (4843 ME) as a potential offspring from ‘Clemens’ and ‘Reinette de Breda Grise’ since both were inferred as offspring of ‘Reinette Franche’ itself on the basis of documented date. In all these 9 cases, the moderate value of ME could therefore be explained by true direct relationships that were incorrectly oriented, together with the possible occurrence of inbreeding. This further highlights the level of complexity in parts of the domesticated apple population. Altogether, the threshold of 600 ME for inferring true complete parent-offspring trios was again considered as very robust and stringent.

Finally, for the potential grandparents-parent-offspring groups, the gap at around 100 ME appeared to constitute a robust inference threshold. Indeed, the two least supported cases with either 85 or 63 ME again involved members of known pedigrees derived from the wild species *Malus floribunda* (‘F2–26829–2-2’ and ‘PRI14–126’) and putatively generating null alleles. On the other side of the threshold, all 6 cases with ME ranging from 312 to 416 consisted of groups with ‘Cox’s Orange Pippin’ as either an offspring of ‘Margil’ or ‘Winston’, with a pairing of ‘Cox’s Orange Pippin’s documented or inferred offspring (‘Fairie Queen’, ‘Prinses Beatrix’, ‘Fiesta’ …) as both grandparents, which is not possible.

### Choice of preferred names for MUNQ

The relationships inferred in this study are based on genotypes preserved in European germplasm collections. Thus the historical and patrimonial value of our findings will rely on the adequate attribution of preferred cultivar names to the MUNQ genotypes (see Methods). We are aware that many synonyms are known and documented for well-known cultivars such as ‘Borowitzky’ = ‘Charlamowsky’ = ‘Duchess of Oldenburg’, or ‘Dutch Mignonne’ = ‘Reinette de Caux’, and additional putative synonyms have been identified recently through the genotyping work performed in numerous germplasm collections (e.g. [32]). Erroneous accession names have been frequently observed in germplasm collections in the past [51]. In the present study however, we focused on well curated collections and considered all available genotypic and passport information in our attribution of the preferred names. This work is likely to evolve in the future with new genotypic, passport and pomological data and no doubt, further questions may be raised regarding the trueness-to-type of some accessions. For example, we found that the parents inferred for accession 1948-737 from the NFC collection (MUNQ 1973), named ‘Topaz’, did not correspond to the recorded pedigree for this cultivar. Thanks to SSR genotyping (data not shown), the accession was also found to be different from another accession with the same name (MUNQ 1213). Thorough examination of collection records showed that ‘Topaz’ is a homonym that has been applied to at least two different cultivars historically. Thus an indication of their origin added to the name would enable an easier distinction between the two. Nonetheless, we consider the parentage assignation in this study to be robust with respect to the genetic material in question, and with respect to the majority of the well-known and well documented cultivars.

### Possible influence of sampling bias

The power of any parentage analysis depends on the size and completeness of the set of genotypes studied [23]: logically, the larger the better. If a particular parent or grandparent is lacking in the studied sample, pedigree reconstruction will be hampered and the number of generations or connections limited, especially if the individual played a critical role within the overall pedigree. In our study, we started from a large sample of over 1400 different genotypes. Based on previous SSR genotyping and analysis [32], choice of SNP-genotyped individuals was optimized to avoid redundancy and to cover a wide genetic diversity of mostly European dessert apple cultivars. Using available SSR data, additional accessions were chosen as likely to be influential in apple pedigrees. This was the case for ‘Reinette Franche’ and ‘Calville Rouge’ identified after a close scrutiny of SSR allele sharing, highlighting the high frequency of their respective alleles in the INRA collection (J.L. Crépin, personal communication). One great advantage of a perennial and clonally-propagated species like apple is the availability of ancient cultivars, some being centuries old. This is a major and extremely favorable point for multi-generation pedigree reconstruction [18].

Nonetheless, within our sample of over 1400 genotypes, the representation of European cultivars was still somewhat unbalanced for various practical and organizational reasons. Although some cultivars were added later to improve the representation of very old or European-wide or even US germplasm, the initial purpose of the genotyping was to perform GWAS with phenotypic data from several sites. The sampling was consequently biased towards cultivars from France, Belgium, United Kingdom, Sweden, Italy and the Czech Republic. Important founders or members of the overall pedigree have almost certainly been overlooked due to the lack of e.g. crucial German or Spanish cultivars. Conversely, the large connectivity networks for cultivars from Great Britain and France were probably facilitated by the strong representation of these countries. Further analyses are likely to reveal equivalent networks from other regions.

As a way to bypass missing parents in the pedigree construction, we attempted to infer grandparents as a replacement for the missing parent in predicted parent-offspring duos. This approach proved to be successful, although in only a rather limited number of situations (Additional file 7: Table S6). One interesting case was nevertheless apparent through the recurrent identification of two jointly-assigned grandparents for 5 inferred offspring, namely ‘Keswick Codlin’ and ‘Hawthornden’ as inferred grandparents for ‘S.T. Wright’, ‘Carlisle Codlin (of Bultitude)’, ‘Cutler Grieve’, ‘Grimoldby Golden’, and ‘Reverend W. Wilks’.

### Consistency of inferred pedigrees with documented dates and parentage

A large proportion of the inferred pedigrees was in agreement with the dates and parentage documented in various pomological books and sources (Additional file 4). This further supports our use of the technique and adds further robustness to the findings. Inconsistencies may arise from either inaccurate documentation or inaccurate pomological identification, and as described above, it is possible that some of the preferred names might be questioned on the basis of our findings.

In many cases, our findings were highly consistent with previously published, marker-based pedigree inferences, such as: the trio involving ‘Geheimrat Doktor Oldenburg’ and ‘Cox’s Orange Pippin’ as the parents of ‘Dukat’ [51]; the parent-offspring duo for ‘Grimes Golden’ and ‘Golden Delicious’ [52]; the trio involving ‘Dutch Mignonne’ and ‘White Astrachan’ (synonym ‘Petite Madeleine’) as the parents of ‘Dülmener Rosenapfel’ [39]; the trio involving ‘Abbondanza’ and ‘Decio’ as the parents of ‘Scodellino’ [32]; the trio involving ‘Cox’s Orange Pippin’ and ‘Cox’s Pomona’ as the parents of ‘Ingrid Marie’ [38], and the trios involving ‘Cox’s Orange Pippin’ and ‘Cellini’ as the parents of ‘Laxton’s Pearmain’ and ‘Ellison’s Orange’ [54]. We also confirm that the widely held belief about ‘Ribston Pippin’ being a parent of ‘Cox’s Pomona’ [27] is false as was recently shown by Larsen et al. [38] and, importantly, we add to this that it also appears not to have been a parent of ‘Cox’s Orange Pippin’.

A few inconsistencies are also worth highlighting, such as that ‘Laxton’s Superb’ and ‘Laxton’s Pearmain’, both generally documented as ‘Wyken Pippin’ x ‘Cox’s Orange Pippin’ (or reciprocal cross) [27] were here inferred as deriving from a cross between ‘Cox’s Orange Pippin’ and ‘Cellini’. The same observation was recently made by Ordidge et al. [54] for ‘Laxton’s Pearmain’. This could be due to an erroneous identification of the tree originally used as a parent by Laxton Bros. Another example is ‘Geheimrat Doktor Oldenburg’, found here to derive from the cross ‘Alexander’ x ‘Ananas Reinette’, which is inconsistent with previous findings of ‘Bauman Reinette’ being one of the parents according to SSR [51]. This could be solved when getting DNA sample of the tree considered in the latter study.

### Historical and heritage value of the results obtained

*The pervasive spread of the ‘Reinette Franche’ genome:* The most highly-connected cultivar in our analysis was ‘Reinette Franche’ with 66 parent-offspring duos. ‘Reinette Franche’ also exhibited the highest score for multi-generation offspring with 243 in total, meaning that 18% of the studied genotypes were related to ‘Reinette Franche’. Many of them emanated from its first generation offspring ‘Reinette de Hollande’ (synonym ‘Reinette Carminée de Hollande’; [26]) obtained from a cross with ‘Reinette des Carmes’. Components of the genome of ‘Reinette Franche’ consequently occur all over the inferred pedigree despite the very large number of varieties in Europe. This result is, however, not completely unexpected considering the old pomological literature. The famous French pomologist A. Leroy [26] wrote in 1873 that ‘Reinette Franche’ is the ‘mother of a considerable number of apple varieties’ and cited Charles Estienne [59] who described the ‘Pommes de Renette’ (a synonym), indicating that this variety was probably already 30 years old in 1540 and originated from Normandie in North-Western France, from where it was widely spread; another synonym of ‘Reinette Franche’ is ‘Reinette de Normandie’ [60]. Leroy [26] also cited the German naturalist J. Mayer who indicated a century earlier that the epithet ‘franche’ refers to France and that this cultivar was the origin of numerous French ‘Reinette’ cultivars [61]. ‘Reinette Franche’ appears to have given rise not only to numerous French cultivars, but also to many well-known cultivars in other countries such as ‘King of the Pippins’ or ‘Peasgood’s Nonsuch’ in United Kingdom, ‘Rose de Berne’ and ‘Rose d’Ajoie Blaser’ in Switzerland, ‘Mela del Sangue’ and ‘Mela del Giappone’ in Italy, ‘Président Roulin’ and ‘Grosse Reinette Transparente Lebeau’ in Belgium, ‘Jonathan’ (through ‘Esopus Spitzenburg’) and ‘Melrose’ in the United States, and ‘Democrat’ (through ‘Reinette de Hollande’ as one of the two grandparents of its missing parent) in Australia.

*Other major founders in Europe:* ‘Reinette des Carmes’ (synonym ‘Reinette Rousse’) is considered to originate from France during the seventeenth century [26] and stands out as a major founder for European cultivars through its role as the other parent of ‘Reinette de Hollande’ (together with ‘Reinette Franche’), and resulting links to numerous offspring spanning the fourth to seventh generations in the pedigree founded by ‘Reinette de Saintonge’.

‘Margil’ formed the basis of another very large multi-generation pedigree, especially through its inferred first generation offspring ‘Cox’s Orange Pippin’. ‘Margil’ (synonym ‘Reinette Musquée’, also called ‘Muscadet’) was mentioned by A. Leroy [26] as a very old variety (described in 1608 by Olivier de Serres) originating from Normandie where it was frequently used for apple juice or cider. ‘Margil’ was believed to be extensively propagated in the nursery of Brompton Park (London, UK) in the middle of the eighteenth century [60] and would therefore be a plausible parent for ‘Cox’s Orange Pippin’. ‘Cox’s Orange Pippin’ was, in turn, raised by Richard Cox at Colnbrook Lawn, Slough, Buckinghamshire, UK, in 1825 [27] and was inferred as the parent of 50 first-generation offspring, including a large number of cultivars from the United Kingdom, such as ‘James Grieve’ and ‘Laxton’s Superb’.

The Russian cultivar ‘Alexander’ (synonyms ‘Aport’ or ‘Aporta’; [26, 27]) was identified as another major founder giving rise to more than 100 offspring in a global pedigree of 5 generations. It originated in the late eighteenth century in the region of Moscow, and gave rise to numerous famous cultivars, such as ‘Cellini’, ‘Cox Pomona’ and ‘Peasgood’s Nonsuch’ in UK, ‘Reinette de Landsberg’ in Germany, ‘Signe Tillisch’ in Denmark, ‘Bismarck’ and ‘Democrat’ in Australia, and ‘Wolf River’ in Wisconsin, USA. The latter cultivar being an example of the influence from intentional introduction of Russian cultivars for use in the upper US Great Plains, as discussed by Volk and Henk [62] and Gross et al. [63].

*Disclosure of the parents of emblematic cultivars:* Disclosure of the complete parentage of ‘Ribston Pippin’, one of the most famous cultivars from the UK, is another major output from the present study. ‘Ribston Pippin’ is reported to have been obtained from a seed brought from Rouen (Normandie, France) around 1690 which produced a tree, still visible in 1815, at Ribston Hall, Yorkshire, UK [26, 27]. It was extensively propagated, both in Great Britain and abroad in the 1800s [26]. It was found to be triploid [64] and here we show that its parents are ‘Margil’, which contributed a *2n*-gamete in agreement with Ordidge et al. [54], and ‘Nonsuch Park’. ‘Margil’ is documented from the early seventeenth century, whereas ‘Nonsuch Park’ is only documented as “Described [in] 1831” [27]. This would suggest that the cultivar may have been around for some time before the date of first description, assuming the accession name is correct. Interestingly, ‘Nonsuch Park’ is also inferred as a first generation progeny of ‘Reinette Franche’ so an earlier date of origin would be plausible. ‘Ribston Pippin’ has also been reputed or reported to be the parent of at least 16 cultivars [27]. Seven of these cultivars were included in our sample, and we inferred both parents for two of them, and one parent for the five others. ‘Ribston Pippin’ was, however, never among the identified parents.

Another intriguing disclosure is the parentage of the well-known cultivar ‘White Transparent’ (synonyms ‘Papirovka’, ‘Klarapfel’, ‘Pomme de Revel’ …) from the Baltic States and dated from the mid-1800s [27], the two inferred parents being ‘Aspa’ and ‘St Germain’. Interestingly, ‘Aspa’ is an, as yet, unique genotype conserved at Balsgård, Kristianstad, Sweden. Beside ‘White Transparent’, ‘Aspa’ was inferred as the parent of only one more cultivar, namely ‘Rivers’ Early Peach’ from England. Thus, the contribution of this supposedly local Swedish cultivar appears to be limited in spite of the success of ‘White Transparent’. Conversely, ‘St Germain’ (possible synonym ‘Virginischer Rosenapfel’, [27]) was inferred as the parent of 14 other cultivars, nine of which originate from Sweden, including ‘Spässerud’, ‘Åkerö’, ‘Vitgylling’, and ‘Sandbergs Röda’, with the latter three inferred as full-sibs with the Swedish ‘Grågylling’ as the other parent. The contribution of ‘White Transparent’ to cultivar development through its 16 first- or second-generation offspring is therefore most likely due to favorable alleles inherited from ‘St Germain’ rather than from ‘Aspa’.

The famous New Zealand cultivar ‘Braeburn’, initially discovered as a chance seedling, had long been hypothesized to be an offspring of ‘Lady Hamilton’ based on genetic distance assessed by RFLP and RAPD markers [65]. In our study, ‘Delicious’ and ‘Sturmer’s Pippin’ were inferred as its parents. ‘Lady Hamilton’ was not genotyped with the 480 K array but SSR data indicated that it most probably derives from the same cross (no ME out of 16 SSR; data not shown), which would make it a full-sib of ‘Braeburn’.

Relatively few cultivars can be successfully grown in warm areas with Mediterranean or subtropical climate (Israel, South Africa, and Florida in the US, …) since most cultivars need prolonged exposure to cold temperatures during winter for bud-break and flowering to occur uniformly [66]. The few cultivars that can be collectively referred to as low-chilling requirement (LCR) cultivars include ‘Anna’ and ‘Golden Dorsett’. Here we inferred ‘Golden Dorsett’ to be an offspring of ‘Anna’ and an unknown parent, itself deriving from a cross between ‘Douce de Sfax’, a Tunisian cultivar likely to have been selected for LCR, and ‘Golden Delicious’. This could explain the homozygosity for the LCR-associated haplotype identified by Trainin et al. [66].

*A historical proofreading of apple selection over centuries:* Exploring the pedigree relationships of a large number of both renowned and little-known, old apple cultivars from Europe and abroad, provides a unique opportunity to study how selection has been performed over centuries. Major and minor historical events such as wars, human migration, or societal evolution may have provoked plant material exchange across short or long distances, amplified by the perennial nature of apple allowing the transport of clonal graft wood in addition to seeds. Our results illustrated four main characteristics of apple selection and breeding throughout history: firstly, contribution of the inferred founders and subsequent cultivars to the overall pedigree is highly unbalanced; secondly, number of detected generations was quite low in most parts of the overall pedigree, similar to grape [18]; thirdly, the frequency of cultivars exhibiting inbreeding detectable within the pedigree was extremely low (2.3%) despite the strong influence of ‘Reinette Franche’ and ‘Margil’; fourthly, crosses have often taken place between cultivars from different regions in Europe in agreement with the weak genetic structure and prominent gene flow found at the European level [32]. These points are further discussed in Additional file 13.

In addition, a similarity was often observed between cultivar names of inferred parent and offspring: for example, nine of the cultivars descended from ‘Reinette Franche’ had the word ‘Reinette’ in their name. The attribution of the same name to a parent and its offspring is most likely prompted by the transmittal of eye-catching pomological traits from one generation to the next.

Finally, identification of cultivars with a significant contribution to the overall pedigree could assist in the choice of progenitors for further crosses in modern breeding programs. ‘Reinette Franche’, ‘Margil’ and ‘Alexander’ could be considered for such purposes. Also, crosses between e.g. ‘Reinette Franche’ and ‘Alexander’ or ‘Margil’ and ‘Red Astrachan’, should allow the genetic mapping of numerous very common QTL to be used for further marker assisted breeding purposes. Alternatively, cultivars of restricted importance in the pedigree but inferred as parents of other famous cultivars, could also be considered either to select full-sibs of such a famous cultivar, or simply to increase the frequency of alternative alleles in the current breeding populations.

## Conclusion

Inferring the parents of an apple cultivar requires a large sample of referenced genotypes, efficient molecular markers and adequate methods for analyzing the resulting data. In the present study, availability of over 1400 apple genotypes, previously filtered for genetic uniqueness and providing a broad representation of European germplasm, has been instrumental for the success of pedigree inferences. Even larger analyses should be pursued to further decipher the genetic relationships across conserved cultivars in public and non-public collections in Europe, North-America, and temperate countries of the Southern-Hemisphere and Central-Eastern Asia. The identification of highly-connected cultivars can aid in future whole-genome sequencing strategies where deep sequencing of a reduced number of founders of the network could help with imputing the genotypes in the remaining individuals genotyped at lower density.

The simple exclusion test that we applied proved to be straightforward and efficient as a first approach to infer relationships. In the future, more elaborated approaches such as computation of haplotype sharing [67] should help to extend the pedigree reconstruction when intermediate genotypes have been definitely lost in germplasm collections. Such approaches may help to resolve the apparent gaps between the very old cultivars, putatively dating from the Roman Ages, and those from the Renaissance period, which would illuminate an unprecedented and fascinating historical link given the emblematic status of the apple within human history.

## Methods

### Plant material

A set of 1425 diploid genotypes was used in this study (Additional file 1: Table S1), and each was given a unique genotype code (MUNQ, for Malus UNiQue genotype code) as a development from the FBUNQ code (for FruitBreedomics UNiQue code) described by Urrestarazu et al. [32] based on SSR data. The set largely replicated the panel built for association studies by Urrestarazu et al. [68], but with an addition of almost 160 accessions from the germplasm collections of the National Fruit Collection (NFC, UK), the Research Center of Laimburg (Italy) and the Biological Resources Center RosePom of INRA (France). Two small segregating populations, each containing 46 progenies and their parents (‘Golden Delicious’ (MUNQ 65) x ‘Renetta Grigia di Torriana’ (MUNQ 435) and ‘Fuji’ (MUNQ 318) x ‘Pinova’ (MUNQ 651)) were also included. In addition, eight triploid genotypes were included (Additional file 1: Table S1).

For each genotype in Additional file 1: Table S1, a “preferred” name was given on the basis of documented synonymy, collection listings, websites and reference pomological books, in addition to information about matching accessions (“duplicates”) as described by Urrestarazu et al. [32]. Historical data on the date of origin, first description, introduction, recording or inclusion in collections of cultivars, and documentation on any presupposed parents were collected from sources mentioned in Additional file 4 and indicated in Additional file 1: Table S1. For the sake of simplicity, such information was further referred to as “documented date” or “documented parentage”, respectively. The SSR data obtained by Fernandez-Fernandez [69], Lassois et al. [39] and Urrestarazu et al. [32] were used to allocate the MUNQ of the accessions and the name initially indicated in these papers was sometimes consequently replaced by the “preferred” name in this study. In our interpretations, we mostly refer to genotypes using their preferred name and these preferred names correspond directly to the MUNQ according to Additional file 1: Table S1.

### SNP genotyping

All genotypes were analyzed with the Axiom**®**Apple480K array containing 487,249 SNPs evenly distributed over the 17 apple chromosomes [55]. The sub-set of 275,223 robust SNPs previously selected by Bianco et al. [55] was initially used for analysis. After the first step of parent-offspring analysis (described below), 22,128 SNPs showing a Mendelian error in two or more accepted relationships were further removed from the genotyping data, leaving a total of 253,095 SNPs for further analyses (Additional file 2: Table S2). A random set of 25,310 (i.e., 10%) of these SNPs was selected for a grandparent search (again, as described below). SNP positions were based on the latest version (v1.1) of the apple genome based on the doubled haploid GDDH13 ( [70]; see also https://iris.angers.inra.fr/gddh13/ for the genome browser).

### Parent-offspring relationships

All possible pairings of diploid individuals were analyzed using PLINK (https://www.cog-genomics.org/plink/1.9/ [56]) for computing Identity By Descent (IBD) sharing probabilities, using the ‘PI_HAT’ parameter. The expected value for first degree relatedness is 0.5. A total of 3655 pairings with a PI_HAT value greater than 0.4 were selected before estimating the number of Mendelian errors (ME) based on a hypothesis that the two individuals were parent and offspring: for example, if an individual had an AA SNP score at a given locus and the other individual of the pairing had a BB SNP score at the same locus, this was considered as a Mendelian inheritance error for a parent-offspring relationship. In an attempt to ensure the inclusion of all first degree relationships in the tested set, the PI_HAT threshold of 0.4 was selected to be lower, and therefore more inclusive, than the value of 0.466 indicated by Myles et al. [21] for a similar study performed on grape cultivars. Any pairings showing fewer than 1000 ME (0.36%) when using the initial set of 275,223 SNPs were considered as potential parent-offspring relations (duos). Using the final set of 253,095 SNPs, this error threshold was reduced to 600 ME (0.24%) such that the corresponding parent-offspring duos could be considered with increased confidence.

### Identification of complete parent-offspring trios

For all diploid individuals that were accepted to potentially be involved in two or more parent-offspring relationships, we counted the number of ME for all possible trios that could associate the individual with two potential parents. In addition to errors due to mutually exclusive homozygous SNP scores, ME in complete parent-offspring trios can also be identified when a potential offspring is scored as heterozygous (AB) and both potential parents are scored as homozygous for only one allele, i.e. both AA or both BB. Based on the distribution of ME over all possible trios, groups that showed fewer than 600 ME in the set of 253,095 SNPs were considered as likely complete parent-offspring sets.

### Identification of parent-offspring duos and complete parent-offspring trios involving triploids

The eight triploids were genotyped and analyzed as if they were diploid, i.e. both genotypes AAB and ABB were treated as AB, while AAA and BBB genotypes were treated as AA and BB, respectively. Consequently, we counted ME as for diploids. To identify potential 2*n*-gamete parents, we counted the SNPs that were homozygous in the triploid offspring and heterozygous in the diploid parent, here called “tri-hom/di-het” SNPs, as ME for a parent-offspring relationship. This number would be expected to be close to zero for the parent that contributed a 2*n*-gamete since both alleles should have been passed to the triploid offspring, with the exception of reassortment through crossovers in 2*n*-gametes formed through first division restitution or second division restitution. The potential *n*-gamete parent was inferred as above for diploid genotypes. Since several triploid cultivars were previously considered to be the parents of various diploid cultivars ([27], e.g., “Ribston Pippin” as the parent of “Cox’s Orange Pippin”), we developed the following procedure to challenge such situations. When a potential 2*n*-gamete parent was identified, we examined the dependency of the other individuals suggested to be offspring of the triploid, on the genotype of the potential 2*n*-gamete (grand)parent. In cases where the triploid was heterozygous and its 2*n*-gamete parent was homozygous we counted: i) the number of SNPs in the potential offspring that were homozygous for the same allele as the 2*n*-gamete (grand)parent or heterozygous, and ii) the number of SNPs in the potential offspring that were homozygous for the alternative allele to that of the potential 2*n*-gamete (grand)parent. Absence (or almost absence) of SNPs in the second category indicated that the supposed triploid intermediary did not pass any alleles received from the other (*n*-gamete) parent to the potential offspring, and thus the triploid could be excluded as a potential parent of this individual.

### Orientation of parent-offspring duos and integration of historical data

For all pairings of diploid individuals inferred to be in a parent-offspring duo that could not be identified as part of a trio, we attempted to determine which individual was the parent and which individual was the offspring. We considered first, that any individual identified as an offspring in a trio would have to be the parent in any other duos that it was involved in; the second individual of the duo was thus considered an offspring. Subsequently, any offspring identified in this way could only be considered a parent in further duos, since the other individual would otherwise have been expected to be identified as its other parent in a trio. The pedigrees were thus progressively constructed according to this iterative process.

We then used historical data to orient additional duos: if one individual in a duo had already been documented as the offspring of the other individual, we assumed that this was probably the case. Where documented dates could be found for both individuals in a duo, we considered that the one with the most recent date was most probably the offspring. The same iterative process was then applied to orient further additional duos for which neither previously reported parentage, nor date of origination enabled orientation.

### Identification of grandparent couples for parent-offspring duos

For each parent-offspring duo that was not identified as part of a trio, the two potential parents of the missing parent, i.e. the grandparent couple were identified, where possible. To do this, we considered as ME those SNPs where both potential grandparents scored as homozygous for a given allele and: (i) the offspring was scored as homozygous for the alternate allele, or (ii) the offspring was scored as heterozygous and the accepted parent was scored as homozygous for the same allele as the potential grandparents. We used a random set of 25,310 SNPs in order to reduce computation time and retained potential grandparent pairings that reported fewer than 100 ME (0.40%) only. Subsequently, we further checked the groups potentially consisting of a grandparent couple, parent and offspring by counting the number of ME in the set of 253,095 SNPs. Finally, groups with a grandparent couple, parent and offspring that reported fewer than 100 ME in the set of 253,095 SNPs (0.04%) were considered likely grandparents-parent-offspring sets. The segregating populations and triploids were excluded from this process.

### Pedigree deduced from all results

All of the inferred trios, oriented duos and groups of grandparents-parent-offspring were used to produce a large pedigree file which could be browsed using the software Pedimap [57]. Again, the two segregating populations were not included in this pedigree.

### Software

ME counts were performed with R-scripts which have been deposited on SourceSup (https://sourcesup.renater.fr/projects/outbredpedigree/). The R package “snpStats” [71] was used to upload the SNP data in R. The package “network” [72] was used to generate Fig. 2. The rosnerTest used to identify outliers for heterozygosity belongs to the package “EnvStats” [73].

## Supplementary information

**Additional file 1: Table S1.** List of the 1433 unique *Malus domestica* individuals (MUNQ) involved in the study, with the accession number of the accession used to represent the MUNQ in genotyping (Accession code), the preferred name attributed to the MUNQ (Preferred Name), and its short version (Preferred Name Short), the original reason for genotyping the MUNQ (Origin), their ploidy level (Ploidy), the date of origin, first description, introduction, recording or inclusion in collections of cultivars corresponding to the preferred name (Date), and reference(s) enabling to document the date (Source for date).

**Additional file 2: Table S2.** List of the 253,095 robust SNPs used in the study, with their chromosome location, and position on chromosome, in bp. SNPs located on chromosome 18 are those located on contigs or scaffold that could not be attributed to a chromosome. SNPs located on chromosome 19 are those for which no clear position could be found (either no BLAST results for the probe, or several equivalent results). Inclusion of a SNP in the 25 K subset is indicated by a “yes” in column “in.25 K.subset”.

**Additional file 3: Table S3.** Complete parent-offspring trios inferred with less than 600 Mendelian Errors (ME), indicating the preferred name of the offspring (Preferred Name Offspring) and its MUNQ (MUNQ O), the preferred names of the parents (Preferred Name P1, Preferred Name P2) and their MUNQ (MUNQ P1, MUNQ P2), the number of ME (# ME), presupposed parents (Parent1 from Literature, Parent 2 from Literature) and source of this documentation (Source_parents), consistency of inferred parents with literature (P1_confirmed and P2_confirmed), consistency of dates, i.e. offspring younger than its inferred parent(s), on the basis of documented dates indicated in Additional file 1: Table S1 (Date consistent P1 and Date consistent P2). The trios for which presupposed parent(s) were identified are listed in the first 143 lines, while the remaining are below. In each part, the trios are sorted alphabetically according to the preferred name of the offspring. When presupposed parents were identified, parents considered as P1 and P2 were sorted according to literature. For the remaining trios, parents were attributed to P1 and P2 arbitrarily.

**Additional file 4.** List of sources used to identify date of origin, first description, introduction, recording or inclusion in collections, or parents according to preferred name.

**Additional file 5: Table S4.** Oriented Parent-Offspring duos inferred with less than 600 Mendelian Errors (ME), indicating the preferred name of the offspring (Preferred Name Offspring) and its MUNQ (MUNQ O), the preferred name of the parent (Preferred Name P) and its MUNQ (MUNQ P), the number of ME (# ME), presupposed parents of the offspring (Parent1 from Literature, Parent 2 from Literature) and source of this documentation (Source_parents), consistency of inferred parent with literature (one parent confirmed), consistency of dates, i.e. offspring younger than its inferred parent, on the basis of documented dates indicated in Additional file 1: Table S1 (Date consistent). The duos for which presupposed parents were identified are listed in the first 25 lines, while the remaining are below. In each part, the duos are sorted alphabetically according to the preferred name of the offspring.

**Additional file 6: Table S5.** Non-Oriented Parent-Offspring duos inferred with less than 600 Mendelian Errors (ME), indicating the preferred name of the two individuals of the pair (Preferred Name Ind1, Preferred Name Ind2) and their MUNQ (MUNQ Ind1, MUNQ Ind2, the number of ME (# ME), MUNQ of supposed offspring (Supposed Offspring) and supposed parent (Supposed parent) in the pair when available with indication of the source of the supposed orientation (oriented using), presupposed parents of both individuals (Ind1_Parent1 from Literature, Ind1_Parent 2 from Literature, Ind2_Parent1 from Literature, Ind2_Parent 2 from Literature) and source of these documentation (Source_parents_ind1, Source_parents_ind2), documented dates of ind1 and ind2 as indicated in Additional file 1: Table S1, and indication of possibility of orientation according to date (Date consistency). The duos which were oriented based on documented parentage (ped-lit in column “oriented using”) are listed in the first 44 lines, and are sorted alphabetically according to the preferred name of the supposed offspring. The duos which were oriented based on documented dates of the two individuals are listed in the next 161 lines and are sorted from the oldest to the most recent supposed parent; next are listed the 43 duos which were oriented because one individual of the duos was supposed a child in a duo oriented according to previously documented parentage or dates; these are sorted from the oldest to the most recent supposed parent; next are listed two duos which were oriented in a second round. Next are listed 51 duos for which a date was listed for one individual only: this individual was then attributed to be Ind2 and all these duos are sorted from the oldest to the most recent Ind2. Next are listed seven duos for which a date was available for both individuals but was too imprecise or too old to enable orientation. The remaining 22 duos for which neither dates nor parentage documentation were found, are listed at the end; individuals that appeared most frequently in these duos were attributed to be Ind2 and all these duos are sorted alphabetically according to the preferred name of Ind2.

**Additional file 7: Table S6.** Groups of grandparents, parent and offspring inferred with less than 100 Mendelian Errors (ME), indicating the preferred names of the offspring (Offspring), parent (Parent) and grandparents (GrandParent 1, GrandParent 2), i.e. parent couple of the missing parent, and their MUNQ (MUNQ O, MUNQ P, MUNQ GP1, MUNQ GP2), the number of ME in the 25 K SNP set (# ME 25 K) and in the 253 K SNP set (# ME 253 K), presupposed parents of the offspring (Parent1 from Literature, Parent 2 from Literature) and source of this documentation (Source_parents), consistency of inferred parent with literature (Ped_consistency), consistency of dates, i.e. offspring younger than its inferred grandparent(s), on the basis of documented dates indicated in Additional file 1: Table S1 (Date consistent GP1 and Date consistent GP2).

**Additional file 8.** Pedigree deduced from all relationships between diploid individuals inferred in the present work. The file can be opened using the Pedimap software [57]. Cultivar names are in their short version (Additional file 1: Table S1). The “indic” column indicates the level of information known for the individual(s) in the pedigree: 1, founders; 2, unknown individuals, with both parents known; 3, semi-founders (one parent known, one parent unknown); 4, known individuals with both parents known. (ZIP 13 kb)

**Additional file 9: Figure S1.** Pedigrees linking the founder ‘Calville Rouge’ to all its offspring, over three generations. Cultivar names are in their short version (see Additional file 1: Table 1). The coloring of the name bars indicates the level of information known for the individual(s) in the pedigree: blue, individuals with both parents known; cream, individuals with one parent known; orange, unknown individual with both parents known; dark green, founders. Relationships are represented by black lines as the mother and the father cannot be identified with our data. The figure was drawn using data extracted from Additional file 8 and the Pedimap software [57]

**Additional file 10: Figure S2.** Distribution of heterozygosity in 1425 diploid individuals.

**Additional file 11: Figure S3.** Distribution of Mendelian Error (ME) counts in 10,720 pairs of diploid-triploid *Malus domestica* individuals tested as parent-offspring duos. The inferred parent-offspring pairings involving the parent giving a diploid gamete to the triploid offspring are accounted for in light gray bars (inf.UNG par-O duos), inferred parent-offspring pairings involving the parent giving a haploid gamete to the triploid offspring in dark gray bars (inf. haplG par-O duos), the full-sib pairings in pink bars (FS), the half-sib pairings through the parent giving a diploid gamete to the triploid offspring in light blue bars (HS via UNG par.), the half-sib or grand-parent-grand-child relations through the parent giving a diploid gamete to the triploid offspring in medium blue bars (HS or GP-GC via UNG par.), the half-sib pairings through the parent giving a haploid gamete to the triploid offspring in blue bars (HS via haplG par.), the half-sib or grand-parent-grand-child relations through the parent giving a haploid gamete to the triploid offspring in dark blue bars (HS or GP-GC via haplG par.),and other pairings in purple bars (unknown). On the left, all tested pairs are represented. On the right pairs with less than 500 ME are shown.

**Additional file 12: Table S7.** Relationships identified for triploids inferred with less than 300 Mendelian Errors (ME) for duos and trios, and less than 1200 tri-hom/di-het with the parent giving a diploid gamete, indicating the preferred name of the triploid offspring (Triploid Offspring) and its MUNQ (MUNQ O), the preferred name of the parent giving a diploid gamete (2*n*-gamete Parent) and its MUNQ (MUNQ 2n P), the number of ME for the duo triploid-2*n*-gamete parent (#ME 2n P), the number of tri-hom/di-het SNPs for the duo triploid-2n-gamete parent (# Hom-Het), the preferred name of the parent giving a haploid gamete (n-gamete Parent) and its MUNQ (MUNQ n P), the number of ME for the duo triploid-n-gamete parent (#ME n P), and when available, the number of ME for the trio (# ME trio).

**Additional file 13.** Additional points of discussion regarding the historical and heritage value of the results obtained

## Data Availability

All SNP genotyping data used in the current study have been deposited in the INRA dataset Archive (https://data.inra.fr/) at 10.15454/IOPGYF.

## Abbreviations

ME: Mendelian errors
MUNQ: Malus UNiQue genotype code
NFC: National Fruit Collection, UK
SNPs: Single nucleotide polymorphisms
SSR: Simple sequence repeat or microsatellite

## References

[1] Visscher PM, Hill WG, Wray NR (2008). Heritability in the genomics era - concepts and misconceptions. Nat Rev Genet.

[2] Visscher PM, Medland SE, Ferreira MAR, Morley KI, Zhu G, Cornes BK (2006). Assumption-free estimation of heritability from genome-wide identity-by-descent sharing between full siblings. PLoS Genet.

[3] Bérénos C, Ellis PA, Pilkington JG, Pemberton JM (2014). Estimating quantitative genetic parameters in wild populations: a comparison of pedigree and genomic approaches. Mol Ecol.

[4] Fernández J, Villanueva B, Pong-Wong R, Toro MÁ (2005). Efficiency of the use of pedigree and molecular marker information in conservation programs. Genetics..

[5] Lucena-Perez M, Soriano L, López-Bao JV, Marmesat E, Fernández L, Palomares F (2018). Reproductive biology and genealogy in the endangered Iberian lynx: implications for conservation. Mamm Biol.

[6] Bink MCAM, Jansen J, Madduri M, Voorrips RE, Durel C-E, Kouassi AB (2014). Bayesian QTL analyses using pedigreed families of an outcrossing species, with application to fruit firmness in apple. Theor Appl Genet.

[7] Bink MCAM, Boer MP, ter Braak CJF, Jansen J, Voorrips RE, van de Weg WE (2008). Bayesian analysis of complex traits in pedigreed plant populations. Euphytica..

[8] Peace CP, Luby JJ, van de WWE, MC a. M B, Iezzoni AF (2014). A strategy for developing representative germplasm sets for systematic QTL validation, demonstrated for apple, peach, and sweet cherry. Tree Genet Genomes.

[9] Dussault FM, Boulding EG (2018). Effect of minor allele frequency on the number of single nucleotide polymorphisms needed for accurate parentage assignment: a methodology illustrated using Atlantic salmon. Aquac Res.

[10] McClure MC, McCarthy J, Flynn P, McClure JC, Dair E, O’Connell DK (2018). SNP data quality control in a National Beef and dairy cattle system and highly accurate SNP based parentage verification and identification. Front Genet.

[11] Calus MP, Mulder HA, Bastiaansen JW (2011). Identification of Mendelian inconsistencies between SNP and pedigree information of sibs. Genet Sel Evol.

[12] Hickey JM, Cleveland MA, Maltecca C, Gorjanc G, Gredler B, Kranis A, Gondro C, van der Werf J, Hayes B (2013). Genotype Imputation to Increase Sample Size in Pedigreed Populations. Genome-Wide Association Studies and Genomic Prediction.

[13] Ellstrand NC (1984). Multiple paternity within the fruits of the wild radish, *Raphanus sativus*. Am Nat.

[14] Gowaty PA, Karlin AA (1984). Multiple maternity and paternity in single broods of apparently monogamous eastern bluebirds (*Sialia sialis*). Behav Ecol Sociobiol.

[15] Burke T, Bruford MW (1987). DNA fingerprinting in birds. Nature..

[16] Jeffreys AJ, Morton DB (1987). DNA fingerprints of dogs and cats. Anim Genet.

[17] Nybom H, Schaal BA (1990). DNA “fingerprints” applied to paternity analysis in apples (*Malus* x *domestica*). Theor Appl Genet.

[18] Lacombe T, Boursiquot J-M, Laucou V, Vecchi-Staraz MD, Péros J-P, This P (2013). Large-scale parentage analysis in an extended set of grapevine cultivars (*Vitis vinifera* L.). Theor Appl Genet.

[19] Testolin R, Marrazzo T, Cipriani G, Quarta R, Verde I, Dettori MT (2000). Microsatellite DNA in peach (*Prunus persica* L. Batsch) and its use in fingerprinting and testing the genetic origin of cultivars. Genome..

[20] Howard NP, van de Weg E, Bedford DS, Peace CP, Vanderzande S, Clark MD (2017). Elucidation of the ‘Honeycrisp’ pedigree through haplotype analysis with a multi-family integrated SNP linkage map and a large apple (*Malus* × *domestica*) pedigree-connected SNP data set. Hortic Res.

[21] Myles S, Boyko AR, Owens CL, Brown PJ, Grassi F, Aradhya MK (2011). Genetic structure and domestication history of the grape. Proc Natl Acad Sci.

[22] Telfer EJ, Stovold GT, Li Y, Silva-Junior OB, Grattapaglia DG, Dungey HS (2015). Parentage reconstruction in *Eucalyptus nitens* using SNPs and microsatellite markers: a comparative analysis of marker data power and robustness. PLoS One.

[23] Jones AG, Small CM, Paczolt KA, Ratterman NL (2010). A practical guide to methods of parentage analysis. Mol Ecol Resour.

[24] Cornille A, Gladieux P, Smulders MJM, Roldán-Ruiz I, Laurens F, Le Cam B (2012). New insight into the history of domesticated apple: secondary contribution of the European wild apple to the genome of cultivated varieties. PLoS Genet.

[25] Juniper BE, Mabberley DJ (2006). The story of the apple [internet].

[26] Leroy A (1873). Dictionnaire de pomologie: contenant l’histoire, la description, la figure des fruits anciens et des fruits modernes les plus généralement connus et cultivés.

[27] Smith M (1971). National Apple Registry of the United Kingdom.

[28] Morgan J, Richards A (2002). The new book of apples: the definitive guide to apples, including over 2000 varieties. Ebury.

[29] Roach FA. Cultivated fruits of Britain: their origin and history. Oxford: Blackwell; 1985. p. 349.

[30] Gross BL, Henk AD, Richards CM, Fazio G, Volk GM (2014). Genetic diversity in *Malus* × *domestica* (Rosaceae) through time in response to domestication. Am J Bot.

[31] Noiton DAM, Alspach PA (1996). Founding clones, inbreeding, Coancestry, and status number of modern apple cultivars. J Am Soc Hortic Sci.

[32] Urrestarazu J, Denancé C, Ravon E, Guyader A, Guisnel R, Feugey L (2016). Analysis of the genetic diversity and structure across a wide range of germplasm reveals prominent gene flow in apple at the European level. BMC Plant Biol.

[33] Volk GM, Bramel P (2017). A strategy to conserve worldwide apple genetic resources: survey results. Acta Hortic.

[34] Way RD, Aldwinckle HS, Lamb RC, Rejman A, Sansavini S, Shen T (1991). APPLES (MALUS). Acta Hortic.

[35] Bühlmann A, Gassmann J, Ingenfeld A, Hunziker K, Kellerhals M, Frey JE (2015). Molecular characterisation of the Swiss fruit genetic resources. Erwerbs-Obstbau..

[36] Ferreira V, Ramos-Cabrer AM, Carnide V, Pinto-Carnide O, Assunção A, Marreiros A (2016). Genetic pool structure of local apple cultivars from Portugal assessed by microsatellites. Tree Genet Genomes.

[37] Garkava-Gustavsson L, Kolodinska Brantestam A, Sehic J, Nybom H (2008). Molecular characterisation of indigenous Swedish apple cultivars based on SSR and S-allele analysis. Hereditas..

[38] Larsen B, Toldam-Andersen TB, Pedersen C, Ørgaard M (2017). Unravelling genetic diversity and cultivar parentage in the Danish apple gene bank collection. Tree Genet Genomes.

[39] Lassois L, Denancé C, Ravon E, Guyader A, Guisnel R, Hibrand-Saint-Oyant L (2016). Genetic diversity, population structure, parentage analysis, and construction of Core collections in the French apple Germplasm based on SSR markers. Plant Mol Biol Report.

[40] Liang W, Dondini L, Franceschi PD, Paris R, Sansavini S, Tartarini S (2015). Genetic diversity, population structure and construction of a Core collection of apple cultivars from Italian Germplasm. Plant Mol Biol Report.

[41] Marconi G, Ferradini N, Russi L, Concezzi L, Veronesi F, Albertini E (2018). Genetic characterization of the apple Germplasm collection in Central Italy: the value of local varieties. Front Plant Sci.

[42] Patzak J, Paprštein F, Henychová A, Sedlák J, Somers D (2012). Comparison of genetic diversity structure analyses of SSR molecular marker data within apple (*Malus* × *domestica*) genetic resources. Genome..

[43] Pina A, Urrestarazu J, Errea P (2014). Analysis of the genetic diversity of local apple cultivars from mountainous areas from Aragon (northeastern Spain). Sci Hortic.

[44] Potts SM, Han Y, Khan MA, Kushad MM, Rayburn AL, Korban SS (2012). Genetic diversity and characterization of a Core collection of *Malus* Germplasm using simple sequence repeats (SSRs). Plant Mol Biol Report.

[45] van Treuren R, Kemp H, Ernsting G, Jongejans B, Houtman H, Visser L (2010). Microsatellite genotyping of apple (*Malus* × *domestica* Borkh.) genetic resources in the Netherlands: application in collection management and variety identification. Genet Resour Crop Evol.

[46] Urrestarazu J, Miranda C, Santesteban L, Royo J (2012). Genetic diversity and structure of local apple cultivars from northeastern Spain assessed by microsatellite markers. Tree Genet Genomes.

[47] Vanderzande S, Micheletti D, Troggio M, Davey MW, Keulemans J (2017). Genetic diversity, population structure, and linkage disequilibrium of elite and local apple accessions from Belgium using the IRSC array. Tree Genet Genomes.

[48] Pereira-Lorenzo S, Urrestarazu J, Ramos-Cabrer A (2017). M., Miranda C, Pina a, Dapena E, et al. analysis of the genetic diversity and structure of the Spanish apple genetic resources suggests the existence of an Iberian genepool. Ann Appl Biol.

[49] Baric S, Storti A, Hofer M, Dalla VJ (2012). Resolving the Parentage of the Apple Cultivar ‘Meran. Erwerbs-Obstbau..

[50] Cabe PR, Baumgarten A, Onan K, Luby JJ, Bedford DS (2005). Using microsatellite analysis to Verify breeding records: a study of `Honeycrisp’ and other cold-hardy apple cultivars. HortScience..

[51] Evans K, Patocchi A, Rezzonico F, Mathis F, Durel C, Fernández-Fernández F (2011). Genotyping of pedigreed apple breeding material with a genome-covering set of SSRs: trueness-to-type of cultivars and their parentages. Mol Breed.

[52] Salvi S, Micheletti D, Magnago P, Fontanari M, Viola R, Pindo M (2014). One-step reconstruction of multi-generation pedigree networks in apple (*Malus* × *domestica* Borkh.) and the parentage of Golden delicious. Mol Breed.

[53] Pikunova A, Madduri M, Sedov E, Noordijk Y, Peil A, Troggio M (2014). ‘Schmidt’s Antonovka’ is identical to ‘common Antonovka’, an apple cultivar widely used in Russia in breeding for biotic and abiotic stresses. Tree Genet Genomes.

[54] Ordidge M, Kirdwichai P, Baksh MF, Venison EP, Gibbings JG, Dunwell JM (2018). Genetic analysis of a major international collection of cultivated apple varieties reveals previously unknown historic heteroploid and inbred relationships. PLoS One.

[55] Bianco L, Cestaro A, Linsmith G, Muranty H, Denancé C, Théron A (2016). Development and validation of the axiom®Apple480K SNP genotyping array. Plant J.

[56] Purcell S, Neale B, Todd-Brown K, Thomas L, Ferreira MAR, Bender D (2007). PLINK: a tool set for whole-genome association and population-based linkage analyses. Am J Hum Genet.

[57] Voorrips RE, Bink MCAM, van de Weg WE (2012). Pedimap: software for the visualization of genetic and phenotypic data in pedigrees. J Hered.

[58] Ronald WG, Temmerman HJ (1982). Tree fruits for the prairie provinces.

[59] Seminarium EC (1540). et plantarium fructiferarum praesertim arborum quae post hortos conseri solent. . R. stephani (Parisiis).

[60] Hogg R (1859). The apple and its varieties.

[61] Mayer J. In: Winterschmidt AW, editor. Pomona Franconica oder natürliche Abbildung und Beschreibung der besten und vorzüglichsten Europäischen Gattungen der Obstbäume und Früchte welche in dem Hochfürstlichen Hofgarten zu Würzburg gezogen werden. Nuremberg: Winterschmidt; 1779. Available from: https://books.google.fr/books?id=S4BitAEACAAJ.

[62] Volk GM, Henk AD (2016). Historic American apple cultivars: identification and availability. J Am Soc Hortic Sci.

[63] Gross BL, Wedger MJ, Martinez M, Volk GM, Hale C (2018). Identification of unknown apple (*Malus* × *domestica*) cultivars demonstrates impact local breeding program on cultivar diversity. Genet Resour Crop Evol.

[64] Darlington CD, Moffett AA (1930). Primary and secondary chromosome balance in *Pyrus*. J Genet.

[65] Gardiner SE, Bassett HCM, Madie C, Noiton D (1996). a. M. Isozyme, randomly amplified polymorphic DNA (RAPD), and restriction fragment-length polymorphism (RFLP) markers used to deduce a putative parent for the `Braeburn’ apple. J Am Soc Hortic Sci.

[66] Trainin T, Zohar M, Shimoni-Shor E, Doron-Faigenboim A, Bar-Ya’akov I, Hatib K (2016). A unique haplotype found in apple accessions exhibiting early bud-break could serve as a marker for breeding apples with low chilling requirements. Mol Breed.

[67] Hill WG, White IMS (2013). Identification of Pedigree Relationship from Genome Sharing. G3 Genes Genomes Genet.

[68] Urrestarazu J, Muranty H, Denancé C, Leforestier D, Ravon E, Guyader A (2017). Genome-wide association mapping of flowering and ripening periods in apple. Front Plant Sci.

[69] Fernandez-Fernandez F (2010). Fingerprinting the National Apple & Pear Collections [internet].

[70] Daccord N, Celton J-M, Linsmith G, Becker C, Choisne N, Schijlen E (2017). High-quality de novo assembly of the apple genome and methylome dynamics of early fruit development. Nat Genet.

[71] Clayton D (2014). snpStats: SnpMatrix and XSnpMatrix classes and methods.

[72] Butts C (2008). network: a Package for Managing Relational Data in R. J Stat Softw.

[73] Millard SP (2013). EnvStats: an R package for environmental statistics.

