# Supplementary material for: Using whole-genome SNP data to reconstruct a large multi-generation pedigree in apple germplasm
Source: BMC Plant Biol. 2020 Jan 2;20:2. doi: 10.1186/s12870-019-2171-6 (PMC6941274; doi:10.1186/s12870-019-2171-6)
Supplement: Supplementary file 4 — Additional file 4. List of sources used to identify date of origin, first description, introduction, recording or inclusion in collections, or parents according to preferred name. [file 12870_2019_2171_MOESM4_ESM.docx]

**Using whole-genome SNP data to reconstruct a large multi-generation pedigree in apple germplasm**

Hélène Muranty^1†^, Caroline Denancé^1†^, Laurence Feugey^1^, Jean-Luc Crépin^2^, Yves Barbier^2^, Stefano Tartarini^3^, Matthew Ordidge^4^, Michela Troggio^5^, Marc Lateur^6^, Hilde Nybom^7^, Frantisek Paprstein^8^, François Laurens^1^, Charles-Eric Durel^1^

^1^ IRHS, INRA, Agrocampus-Ouest, Université d'Angers, SFR 4207 QuaSaV, Beaucouzé, France

^2^ Les Croqueurs de Pommes du Confluent Ain-Isère-Savoie, Les Avenières, France

^3^ Department of Agricultural Sciences, University of Bologna, Bologna, Italy

^4^ University of Reading, School of Agriculture, Policy and Development, Whiteknights, Reading, United Kingdom

^5^ Fondazione Edmund Mach, San Michele all'Adige, Trento, Italy

^6^ CRA-W, Centre Wallon de Recherches Agronomiques, Plant Breeding & Biodiversity, Gembloux, Belgium

^7^ Swedish University of Agricultural Sciences, Department of Plant Breeding, Balsgård, Kristianstad, Sweden

^8^ RBIPH, Research and Breeding Institute of Pomology Holovousy Ltd., Horice, Czech Republic

**Supplementary references**

Sources used to identify date of origin, first description, introduction, recording or inclusion in collections, or parents according to preferred name. For data extracted from references 12, 17 and 37, SSR data were used to identify the MUNQ of the accessions for which parentage was indicated and the name indicated in the references was sometimes modified to match the current preferred name used for the MUNQ.

1 Anonymous (1948) Le Verger Français. Fruits locaux et régionaux, Pommes américaines. Société Pomologique de France, Lyon-Paris

2 Baric S, Storti A, Hofer M, Dalla Via J (2012) Resolving the Parentage of the Apple Cultivar ‘Meran.’ Erwerbs-Obstbau 54:143–146. doi: 10.1007/s10341-012-0167-6

3 Beach SA, Booth NO, Rogers B, Taylor OM (1905) The apples of New York /. J.B. Lyon, Albany :

4 Bink MCAM, Jansen J, Madduri M, et al. (2014) Bayesian QTL analyses using pedigreed families of an outcrossing species, with application to fruit firmness in apple. Theor Appl Genet 127:1073–1090. doi: 10.1007/s00122-014-2281-3

5 Bordeianu T, Constantinescu N, Stefan N (1964) Pomologia, Republicii Populare Romine. 2. Marul. Academiei Republicii Socialiste Romania, Bucarest

6 Brooks RM, Olmo HP (1952) Register of new fruit and nut varieties, 1920-1950. University of California Press, Berkeley and Los Angeles

7 Bruvenich F, van Hulle H-J (1868) Excursion pomologique et arboticole à l’exposition universelle et aux environs de Paris. Bulletins du Cercle Professoral pour le Porgrès de l’Arboriculture en Belgique 161–178.

8 Cabe PR, Baumgarten A, Onan K, et al. (2005) Using Microsatellite Analysis to Verify Breeding Records: A study of `Honeycrisp’ and Other Cold-hardy Apple Cultivars. HortScience 40:15–17.

9 Calhoun CL (2011) Old Southern Apples: A Comprehensive History and Description of Varieties for Collectors, Growers, and Fruit Enthusiasts, 2nd Edition. Chelsea Green Publishing,

10 Carmine B, Croq G, Jardillier D, et al. (2016) La collection fruitière du jardin du Luxembourg. Turriers, France

11 Choisel J-L (1996) Guide des Pommes. Ed. Hervas, Paris, France

12 Choisel J-L (2013) Chercheur de pommes.

13 Diel AFA (1826) Systematische Beschreibung der vorzüglichsten in Deutschland vorhandenen Kernobstsorten: Aepfel - Birnen. Cotta, Stuttgart und Tübingen, Germany

14 Eneroth O (1877) Handbok i Svensk pomologi: Andra upplagan. P. A. Norstedt & Söner, Stockholm, Sweden

15 Evans K, Patocchi A, Rezzonico F, et al. (2011) Genotyping of pedigreed apple breeding material with a genome-covering set of SSRs: trueness-to-type of cultivars and their parentages. Molecular Breeding 28:535–547. doi: 10.1007/s11032-010-9502-5

16 Garkava-Gustavsson L, Kolodinska Brantestam A, Sehic J, Nybom H (2008) Molecular characterisation of indigenous Swedish apple cultivars based on SSR and S-allele analysis. Hereditas 145:99–112. doi: 10.1111/j.0018-0661.2008.02042.x

17 Hampson CR, Kemp H (2003) Characteristics of important commercial apple cultivars. In:Ferree DC, Warrington IJ(ed) Apples: botany, production and uses ed. CABI, Wallingford, pp61–89

18 Hennau C-A (1856) Calville Rouge d’Hiver. In:(ed) Annales de pomologie belge et étrangère ed. Parent, Bruxelles, Belgium, pp11–12

19 Hogg R (1859) The apple and its varieties. Groombridge and sons, London, UK

20 Holland D, Bar-Ya’akov I, Hatib K (2005) Apple genetic resources in Israel. Journal of the American Pomological Society 69:186–200.

21 Igarashi M, Hatsuyama Y, Harada T, Fukasawa-Akada T (2016) Biotechnology and apple breeding in Japan. Breeding Science 66:18–33. doi: 10.1270/jsbbs.66.18

22 Larsen B, Toldam-Andersen TB, Pedersen C, Ørgaard M (2017) Unravelling genetic diversity and cultivar parentage in the Danish apple gene bank collection. Tree Genetics & Genomes. doi: 10.1007/s11295-016-1087-7

23 Lassois L, Denancé C, Ravon E, et al. (2016) Genetic Diversity, Population Structure, Parentage Analysis, and Construction of Core Collections in the French Apple Germplasm Based on SSR Markers. Plant Mol Biol Rep 34:827–844. doi: 10.1007/s11105-015-0966-7

24 Leroy A (1873) Dictionnaire de pomologie: contenant l’histoire, la description, la figure des fruits anciens et des fruits modernes les plus généralement connus et cultivés. Imprimerie Lachèse, Belleuvre et Dolbeau, Paris

25 Miller EP, Sherman WB (1980) Origin and Description of “Dorsett Golden” Apple. Proc. Fla State Hort. Soc. 93:108–109.

26 Morgan J, Richards A (2002) The New Book of Apples: The Definitive Guide to Apples, Including Over 2000 Varieties. Ebury,

27 Noiton DAM, Alspach PA (1996) Founding Clones, Inbreeding, Coancestry, and Status Number of Modern Apple Cultivars. J. Amer. Soc. Hort. Sci. 121:773–782.

28 Nybom H (2004) ’Frida’ and ’Fredrik’, the first scab-resistant apple cultivars developed in Sweden. Acta Horticulturae 871–874. doi: 10.17660/ActaHortic.2004.663.157

29 Nybom H, Sehic J, Garkava-Gustavsson L (2008) Self-incompatibility alleles of 104 apple cultivars grown in northern Europe. The Journal of Horticultural Science and Biotechnology 83:339–344.

30 Ordidge M, Kirdwichai P, Baksh MF, Venison EP, Gibbings JG , Dunwell JM (subm.) Genetic analysis of a major international collection of cultivated apple varieties reveals previously unknown historic heteroploid and inbred relationships

31 Patzak J, Paprštein F, Henychová A, Sedlák J (2012) Genetic diversity of Czech apple cultivars inferred from microsatellite markers analysis. Horticultural Sci.(Prague) 39:149-157

32 Pikunova A, Madduri M, Sedov E, et al. (2014) ‘Schmidt’s Antonovka’ is identical to ‘Common Antonovka’, an apple cultivar widely used in Russia in breeding for biotic and abiotic stresses. Tree Genetics & Genomes 10:261–271. doi: 10.1007/s11295-013-0679-8

33 Pynaert E (1874) Revue del’arboriculture et de la Pomologie. Bulletins d’Arboriculture, de Floriculture et de Culture Potagère 168–174.

34 Reim S, Flachowsky H, Hanke M-V, Peil A (2009) Verifying the parents of the Pillnitzer apple cultivars. Acta Horticulturae 319–324. doi: 10.17660/ActaHortic.2009.814.50

35 Rolff JH (2001) Der Apfel - Sortennamen und Synonyme. Books on Demand,

36 Royer A (1854) Pommes de verger. In:(ed) Annales de pomologie belge et étrangère ed. Parent, Bruxelles, Belgium, pp47–49

37 Røen D, Moe S, Nornes L (2000) Early ripening apple cultivars from Norway. Acta Horticulturae 685–688. doi: 10.17660/ActaHortic.2000.538.123

38 Salvi S, Micheletti D, Magnago P, et al. (2014) One-step reconstruction of multi-generation pedigree networks in apple (Malus × domestica Borkh.) and the parentage of Golden Delicious. Mol Breeding 34:511–524. doi: 10.1007/s11032-014-0054-y

39 Seppä L (2014) Domestic apple cultivars: Sensory descriptions and consumer responses. University of Helsinki, Helsinki, Finland

40 Smith M (1971) National Apple Registry of the United Kingdom. Ministry of Agriculture, Fisheries and Food, London, UK

41 Sokolov VV, Savel’ev NI, Goncharov NP (2015) I. V. Michurin’S Work on Expansion of the Plant Horticulture Assortment and Improvement of Food Quality. Proceedings of the Latvian Academy of Sciences. Section B. Natural, Exact, and Applied Sciences. doi: 10.1515/prolas-2015-0028

42 Strik BC, Proctor JTA (1985) Apple cultivars bred in Canada: selections from controlled crosses for commercial production. Fruit Varieties Journal 40:51–55.

43 Urrestarazu J, Miranda C, Santesteban L, Royo J (2012) Genetic diversity and structure of local apple cultivars from Northeastern Spain assessed by microsatellite markers. Tree Genetics & Genomes 8:1163–1180. doi: 10.1007/s11295-012-0502-y

44 Urrestarazu J, Denancé C, Ravon E, et al. (2016) Analysis of the genetic diversity and structure across a wide range of germplasm reveals prominent gene flow in apple at the European level. BMC Plant Biology 16:130. doi: 10.1186/s12870-016-0818-0

45 Yoshida Y (1977) Progress of Apple Breeding in Japan. 11:56–59.

46 Les croqueurs de pommes, Jardin botanique du Ranquet, verger conservatoire de la vallée de la Loire, verger du Vernet (2014) Fruits d'Auvergne. Union Pomologique de France, Versailles, France

47 Les croqueurs de pommes (2016) Fruits de Poitou-Charentes. Union Pomologique de France, Versailles, France

48 Les croqueurs de pommes, le musée départemental du Revermont, association l’œil dormant (in press) Fruits de Rhône-Alpes. Union Pomologique de France, Versailles, France

49 http://1000obstbaeume.de/2015/02/ruhm-von-kirchwerder/

50 http://appleharvester.blogspot.fr/2010/05/story-of-apple-ben-davis_04.html

51 http://articles.extension.org/pages/60916/apple-rootstock-info:-mm106-emla

52 http://ecbrownsnursery.biz/index.cfm/fuseaction/plants.plantDetail/plant_id/10/index.htm

53 http://library.wur.nl/speccol/fruithof/fruit/App/Tekst/AppT53.htm

54 http://plasgard.se/sida-2/paronsorter/gragylling.html

55 http://portale.provincia.vr.it/uffici/uffici/6/603/documenti/sperimentazione-frutticola-e-vitivinicola/progetto-liste-di-orientamento-varietale-dei-fruttiferi/schede-germoplasma-melo/rosa-mantovana/at_download/file

56 http://rwdf.cra.wallonie.be/fr/patrimoine-fruitier/varietes/marie-joseph-doth%C3%A9e-gosselet

57 http://rwdf.cra.wallonie.be/fr/patrimoine-fruitier/varietes/pr%C3%A9sident-roulin

58 http://s.drocourt.free.fr/sitesperso/Eaubonne/Varietes_originales/Varietes_originales.htm

59 http://www.apfel.ch/frameset.aspx?section=hm2&strhm=produkte

60 http://www.ars-grin.gov/npgs/pi_books/scans/204pt1/pi204pt1_325.pdf

61 http://www.dalival.com/pommes/dalinco/

62 http://www.dalival.com/pommes/dalinsweet/

63 http://www.fruit.usask.ca/apples/heyer12.html

64 http://www.fruit.usask.ca/apples/patterson.html

65 http://www.fruit.usask.ca/apples/rescue.html

66 http://www.fruit.usask.ca/apples/trail.html

67 http://www.fruit.usask.ca/apples/Westland.html

68 http://www.hardyfruittrees.ca/catalog/apple-trees/parkland-zone-2a-an-excellent-quality-apple-commercially-grown-in-alaska

69 http://www.karnhuset.com/landskaps.html

70 http://www.nationalfruitcollection.org.uk/index.php

71 http://www.obstgarten.biz/info-thek/baldenheimer-weissapfel.html

72 http://www.obstgarten.biz/info-thek/marmorapfel.html

73 http://www.obstsortendatenbank.de/osdb/eng/himbsels_rambur_eng.pdf

74 http://www.obstsortendatenbank.de/osdb/ih/edelroter_ih.pdf

75 http://www.obstsortendatenbank.de/osdb/ih/roter_jungfernapfel_ih.pdf

76 http://www.obstsortendatenbank.de/osdb/nda/falchs_gulderling_nda.pdf

77 http://www.obstsortendatenbank.de/osdb/nda/roter_herbstkalvill_nda.pdf

78 http://www.orangepippin.com/apples/beacon

79 http://www.orangepippin.com/apples/franklin

80 http://www.orangepippin.com/apples/rubens

81 http://www.pomologie.com/pomme1/fpommes/calvillemontdor/calvillemontdore.html

82 http://www.pomologie.com/pomme1/fpommes/charlesross/charlesross.html

83 http://www.pomologie.com/pomme1/fpommes/landsbergerreinette/reinettelandsberg.html

84 http://www.pomum.fr/?p=18

85 http://www.puresdarzi.lv/portfolio-view/ausma

86 http://www.regione.piemonte.it/agri/area_tecnico_scientifica/biodiversita/dwd/Cultivar_mele_DEF.pdf

87 http://www.sadarstvi.cz/chodske/

88 http://www.sadarstvi.cz/hajkova-reneta/

89 http://www.sonneruplund.dk/eng/html/Grossherzog.html

90 http://www.sonneruplund.dk/eng/html/Sariola.html

91 http://www.sore.fr/Le-village/Histoire-et-patrimoine/Pomme-de-Sore-A380

92 http://www.suttonelms.org.uk/apple52.html

93 http://www2.assemblee-nationale.fr/sycomore/fiche/(num_dept)/5575

94 https://croqueurs-national.fr/vie-de-l-association.html?catid=0&id=98

95 https://cs.wikipedia.org/wiki/Bl%C3%A1hovo_oran%C5%BEov%C3%A9

96 https://de.wikipedia.org/wiki/Altl%C3%A4nder_Pfannkuchenapfel

97 https://de.wikipedia.org/wiki/Gew%C3%BCrzluiken

98 https://de.wikipedia.org/wiki/Nathusius%E2%80%99_Taubenapfel

99 https://en.wikipedia.org/wiki/Annurca

100 https://en.wikipedia.org/wiki/Braeburn

101 https://en.wikipedia.org/wiki/Red_Delicious

102 https://en.wikipedia.org/wiki/Sturmer_Pippin

103 https://en.wikipedia.org/wiki/Winesap

104 https://en.wikipedia.org/wiki/York_Imperial

105 "https://fr.wikipedia.org/wiki/Deltana

"

106 https://fr.wikipedia.org/wiki/Gloster_69

107 https://fructus.ch/wp-content/uploads/2016/12/150310_feldobst_sortenliste_fructus.pdf

108 https://fruitpluktuin.nl/fruit/Appel/gronigerkroon

109 https://grinczech.vurv.cz/gringlobal/accessiondetail.aspx?id=49115

110 https://grinczech.vurv.cz/gringlobal/accessiondetail.aspx?id=49118

111 https://grinczech.vurv.cz/gringlobal/accessiondetail.aspx?id=49122

112 https://grinczech.vurv.cz/gringlobal/accessiondetail.aspx?id=49127

113 https://grinczech.vurv.cz/gringlobal/accessiondetail.aspx?id=49195

114 https://grinczech.vurv.cz/gringlobal/accessiondetail.aspx?id=49198

115 https://grinczech.vurv.cz/gringlobal/accessiondetail.aspx?id=49319

116 https://grinczech.vurv.cz/gringlobal/accessiondetail.aspx?id=49331

117 https://grinczech.vurv.cz/gringlobal/accessiondetail.aspx?id=49375

118 https://grinczech.vurv.cz/gringlobal/accessiondetail.aspx?id=49391

119 https://grinczech.vurv.cz/gringlobal/accessiondetail.aspx?id=49403

120 https://grinczech.vurv.cz/gringlobal/accessiondetail.aspx?id=49405

121 https://grinczech.vurv.cz/gringlobal/accessiondetail.aspx?id=49406

122 https://grinczech.vurv.cz/gringlobal/accessiondetail.aspx?id=49435

123 https://grinczech.vurv.cz/gringlobal/accessiondetail.aspx?id=49466

124 https://grinczech.vurv.cz/gringlobal/accessiondetail.aspx?id=49468

125 https://grinczech.vurv.cz/gringlobal/accessiondetail.aspx?id=49909

126 https://grinczech.vurv.cz/gringlobal/accessiondetail.aspx?id=49501

127 https://grinczech.vurv.cz/gringlobal/accessiondetail.aspx?id=49530

128 https://grinczech.vurv.cz/gringlobal/accessiondetail.aspx?id=49546

129 https://grinczech.vurv.cz/gringlobal/accessiondetail.aspx?id=49637

130 https://grinczech.vurv.cz/gringlobal/accessiondetail.aspx?id=49709

131 https://grinczech.vurv.cz/gringlobal/accessiondetail.aspx?id=49787

132 https://grinczech.vurv.cz/gringlobal/accessiondetail.aspx?id=49808

133 https://grinczech.vurv.cz/gringlobal/accessiondetail.aspx?id=49811

134 https://grinczech.vurv.cz/gringlobal/accessiondetail.aspx?id=49812

135 https://grinczech.vurv.cz/gringlobal/accessiondetail.aspx?id=49889

136 https://grinczech.vurv.cz/gringlobal/accessiondetail.aspx?id=50062

137 https://grinczech.vurv.cz/gringlobal/accessiondetail.aspx?id=50117

138 https://grinczech.vurv.cz/gringlobal/accessiondetail.aspx?id=50129

139 https://grinczech.vurv.cz/gringlobal/accessiondetail.aspx?id=50131

140 https://grinczech.vurv.cz/gringlobal/accessiondetail.aspx?id=50135

141 https://grinczech.vurv.cz/gringlobal/accessiondetail.aspx?id=50139

142 https://grinczech.vurv.cz/gringlobal/accessiondetail.aspx?id=50157

143 https://grinczech.vurv.cz/gringlobal/accessiondetail.aspx?id=50159

144 https://grinczech.vurv.cz/gringlobal/accessiondetail.aspx?id=50161

145 https://grinczech.vurv.cz/gringlobal/accessiondetail.aspx?id=50166

146 https://grinczech.vurv.cz/gringlobal/accessiondetail.aspx?id=50171

147 https://grinczech.vurv.cz/gringlobal/accessiondetail.aspx?id=50173

148 https://grinczech.vurv.cz/gringlobal/accessiondetail.aspx?id=50199

149 https://grinczech.vurv.cz/gringlobal/accessiondetail.aspx?id=50203

150 https://hort.purdue.edu/newcrop/pri/coop38-2.html

151 https://hort.purdue.edu/newcrop/pri/coop43-2.html

152 https://library.wur.nl/speccol/fruitvrij/aepfel/Aepf1/Ae046.htm

153 https://obstsortenerhalt.de/obstart/details/6660

154 https://portal.mtt.fi/portal/page/portal/mtt_en/projects/Nordapp/Apple%20breeding%20and%20varieties%20in%20Finland1.pdf

155 https://www.appelcollecties.nl/detail.asp?appelnr=75516

156 https://www.arboschwin.com/index.php?page=affi_pomme_ad&num=12

157 https://www.arboschwin.com/index.php?page=affi_pomme_mr&num=56

158 https://www.arche-noah.at/files/fey_s_record.pdf

159 https://www.arche-noah.at/files/obstsortenblatt_2016_ilzer_rosenapfel_web.pdf

160 https://www.ars-grin.gov/npgs/pi_books/scans/pi204pt2.pdf

161 https://www.aujardin.info/fiches/pommes-france-environs.php

162 https://www.cchs-nb.ca/html/Sharp-F_P.html

163 https://www.deutsche-genbank-obst.de/passport/index

164 https://www.ecured.cu/Verde_doncella_(Manzana)

165 https://www.fruitiers.net/fiche.php?id=1136

166 https://www.fruitiers.net/fiche.php?id=618

167 https://www.fruitiers.net/fiche.php?NumFiche=3317

168 https://www.genesys-pgr.org/acn/id/4103768

169 https://www.nbd.gov.lv/lv/slavas-zale/skirne/613

170 https://www.nbd.gov.lv/lv/slavas-zale/skirne/615

171 https://www.nbd.gov.lv/lv/slavas-zale/skirne/616

172 https://www.nordgen.org/nak/index.php?view=show&id=7365&chglang=ENG

173 https://www.oberlausitz-stiftung.de/cms/upload/Literaturdateien/Oberlausitzer_Muskatrenette.pdf

174 https://www.pomologen-verein.de/fileadmin/user_upload/Landesgruppen/2014_Gelber_Richard_Beschreibung.pdf

175 https://www.tawi.fi/wiplant/fine/finesamo.html

176 Shigeki Moriya, pers. comm.
