# Supplementary figures and images for: Using whole-genome SNP data to reconstruct a large multi-generation pedigree in apple germplasm
Source: BMC Plant Biol. 2020 Jan 2;20:2. doi: 10.1186/s12870-019-2171-6 (PMC6941274; doi:10.1186/s12870-019-2171-6)

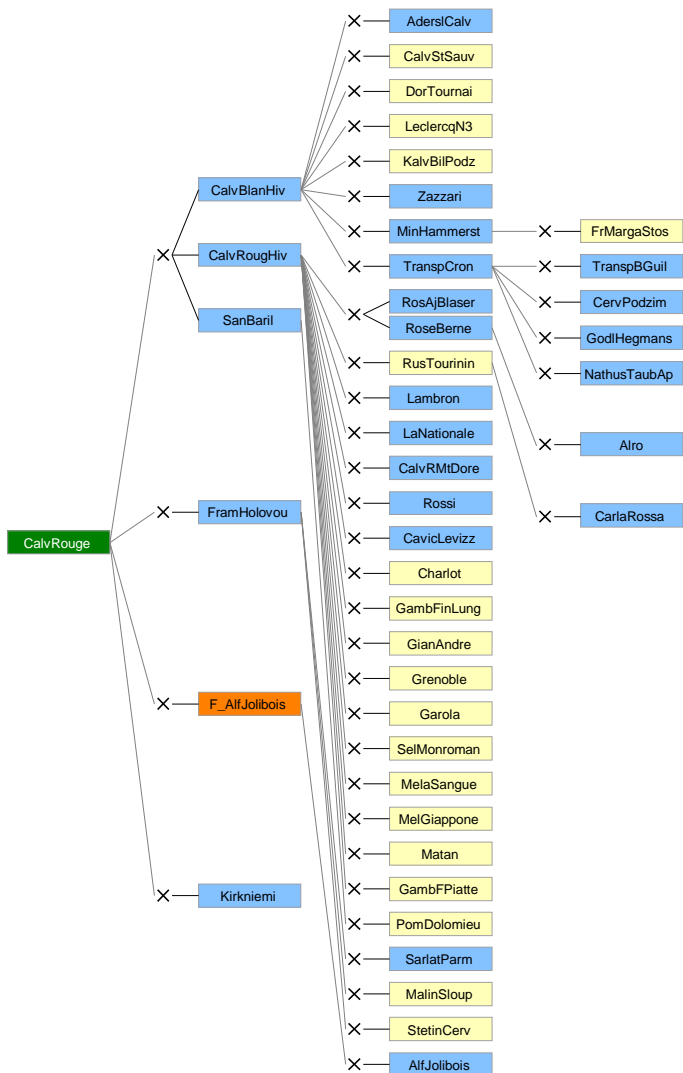

Supplement: Supplementary file 9 — Additional file 9: Figure S1. Pedigrees linking the founder ‘Calville Rouge’ to all its offspring, over three generations. Cultivar names are in their short version (see Additional file 1: Table 1). The coloring of the name bars indicates the level of information known for the individual(s) in the pedigree: blue, individuals with both parents known; cream, individuals with one parent known; orange, unknown individual with both parents known; dark green, founders. Relationships are represented by black lines as the mother and the father cannot be identified with our data. The figure was drawn using data extracted from Additional file 8 and the Pedimap software [57] [file 12870_2019_2171_MOESM9_ESM.pdf]

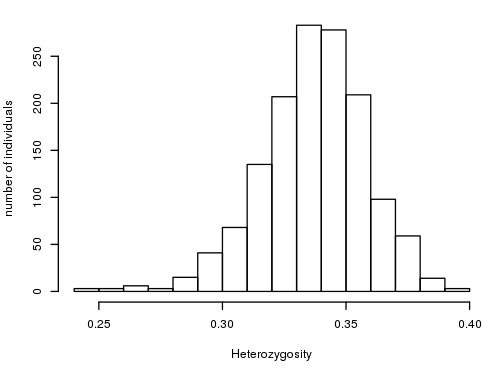

Supplement: Supplementary file 10 — Additional file 10: Figure S2. Distribution of heterozygosity in 1425 diploid individuals. [file 12870_2019_2171_MOESM10_ESM.tif]

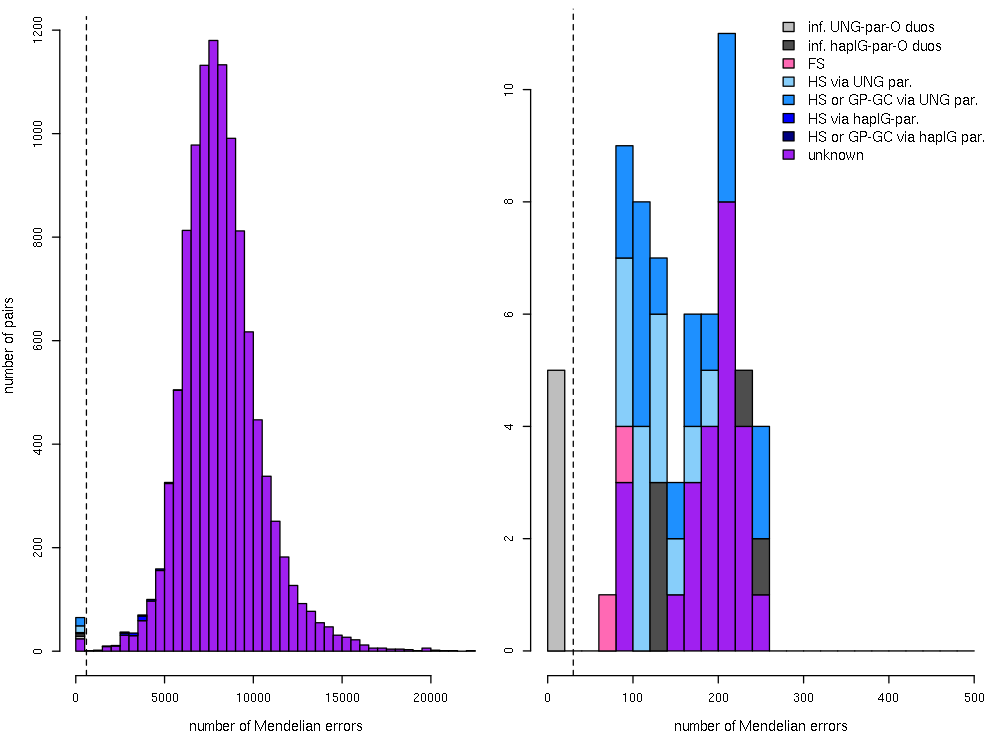

Supplement: Supplementary file 11 — Additional file 11: Figure S3. Distribution of Mendelian Error (ME) counts in 10,720 pairs of diploid-triploid Malus domestica individuals tested as parent-offspring duos. The inferred parent-offspring pairings involving the parent giving a diploid gamete to the triploid offspring are accounted for in light gray bars (inf.UNG par-O duos), inferred parent-offspring pairings involving the parent giving a haploid gamete to the triploid offspring in dark gray bars (inf. haplG par-O duos), the full-sib pairings in pink bars (FS), the half-sib pairings through the parent giving a diploid gamete to the triploid offspring in light blue bars (HS via UNG par.), the half-sib or grand-parent-grand-child relations through the parent giving a diploid gamete to the triploid offspring in medium blue bars (HS or GP-GC via UNG par.), the half-sib pairings through the parent giving a haploid gamete to the triploid offspring in blue bars (HS via haplG par.), the half-sib or grand-parent-grand-child relations through the parent giving a haploid gamete to the triploid offspring in dark blue bars (HS or GP-GC via haplG par.),and other pairings in purple bars (unknown). On the left, all tested pairs are represented. On the right pairs with less than 500 ME are shown. [file 12870_2019_2171_MOESM11_ESM.tif]
