# Supplementary material for: Using whole-genome SNP data to reconstruct a large multi-generation pedigree in apple germplasm
Source: BMC Plant Biol. 2020 Jan 2;20:2. doi: 10.1186/s12870-019-2171-6 (PMC6941274; doi:10.1186/s12870-019-2171-6)
Supplement: Supplementary file 13 — Additional file 13. Additional points of discussion regarding the historical and heritage value of the results obtained [file 12870_2019_2171_MOESM13_ESM.docx]

**Using whole-genome SNP data to reconstruct a large multi-generation pedigree in apple germplasm**

Hélène Muranty^1†^, Caroline Denancé^1†^, Laurence Feugey^1^, Jean-Luc Crépin^2^, Yves Barbier^2^, Stefano Tartarini^3^, Matthew Ordidge^4^, Michela Troggio^5^, Marc Lateur^6^, Hilde Nybom^7^, Frantisek Paprstein^8^, François Laurens^1^, Charles-Eric Durel^1^

^1^ IRHS, INRA, Agrocampus-Ouest, Université d'Angers, SFR 4207 QuaSaV, Beaucouzé, France

^2^ Les Croqueurs de Pommes du Confluent Ain-Isère-Savoie, Les Avenières, France

^3^ Department of Agricultural Sciences, University of Bologna, Bologna, Italy

^4^ University of Reading, School of Agriculture, Policy and Development, Whiteknights, Reading, United Kingdom

^5^ Fondazione Edmund Mach, San Michele all'Adige, Trento, Italy

^6^ CRA-W, Centre Wallon de Recherches Agronomiques, Plant Breeding & Biodiversity, Gembloux, Belgium

^7^ Swedish University of Agricultural Sciences, Department of Plant Breeding, Balsgård, Kristianstad, Sweden

^8^ RBIPH, Research and Breeding Institute of Pomology Holovousy Ltd., Horice, Czech Republic

**Supplementary Data**

**Additional points of discussion regarding the historical and heritage value of the results obtained**

*'Reinette Franche'*: Based on the date for the first description of varieties related to 'Reinette Franche', we identified 'Reinette de Saintonge' as a putative parent of 'Reinette Franche'. 'Reinette de Saintonge' was already described in the 13^th^ century according to Leroy (1), who also mentioned two synonyms 'Blandilalie' and 'Haute-Bonté' as being more frequently used in the previous centuries. Intriguingly, no additional duos were found for 'Reinette de Saintonge' in our data. Additional analysis is necessary to validate the orientation of this parent-offspring relationship.

*'Reinette des Carmes*': Interestingly, in addition to the offspring 'Reinette de Hollande', only two other first generation offspring were inferred, namely 'Reinette de Multhaupt' (synonym “Multhaupts Carmine Reinette” 2) and 'Kilkenny Pearmain'.

*Influence from intentional introduction of Russian cultivars*: Similar to the cultivar 'Alexander', the famous Russian cultivars 'Borowitsky' and 'Red Astrachan' (MUNQ 82), are inferred to have given rise to the well-known North-American cultivars 'Wealthy' and 'Stark Earliest' (MUNQ 468), respectively.

*Around 'Braeburn'*: In addition to being a parent of the full-sibs 'Braeburn' and 'Lady Hamilton', 'Sturmer's Pippin' was inferred to be a progeny of 'Nonpareil' and the parent of two half-sibs of 'Braeburn' and ‘Lady Hamilton’, namely 'Hog's Snout' (MUNQ 1959) and 'Lady Isabel' (MUNQ 1977), both from the UK.

*A historical proofreading of apple selection over centuries*

*Unbalanced contributions to the overall pedigree*: Very old cultivars reputedly dating from the Roman times ('Annurca' and 'Decio') or the Middle Ages ('Martranche', 'Old Pearmain (of Kelsey)', 'Châtaignier' and 'Reinette de Saintonge') either had no offspring in our sample ('Annurca') or had only a single progeny each ('Scodelino', 'Glane', 'Harling Hero', 'Franc Roseau du Valais', and 'Reinette Franche', respectively). The agronomic or organoleptic quality of these very old cultivars was probably insufficient in comparison with the standards of more recent years. The same was observed for some cultivars documented to originate from the Renaissance period (15^th^, 16^th^ and beginning of 17^th^ century) such as 'London Pippin', 'Joaneting', and 'Api Etoilée'. Intriguingly, the only other cultivars documented from this period are the very influential 'Reinette Franche' and its close relatives 'Calville Rouge d'Hiver' and 'Nonpareil', as well as 'Margil’ which has generated numerous multi-generation progenies. Surprisingly, whilst Leroy (1) indicated that 'Reinette Franche' was the 'mother of a considerable number of apple varieties', he did not give any clues to justify such a statement. However, a pomological description of ‘Reinette Franche’ from the INRA germplasm collection indicates a sweet and subacid fruit, with a slight hint of bitterness typical of most old varieties, a firm flesh and a long storage ability (L. Feugey, personal communication). About a century later, another major cultivar showed up from Eastern Europe, namely 'Alexander'.

*Low number of detected generations in most parts of the overall pedigree*: This is consistent with the perennial nature of apple and is similar to what has been described in grape by Lacombe et al. (3). These authors invoked either long-term genetic erosion preventing access to putative older ancestors, or a low number of generations since grape domestication. In the case of apples, the restricted involvement of cultivars from the Middle Ages in the overall pedigree can be considered as an indication of the absence of intermediate genitors preventing the reconstruction of a complete pedigree.

*Extremely low inbreeding detectable within the pedigree*: Empirical and intentional breeding, together with the self-incompatibility system, have probably helped to avoid genotypes that suffer from an adverse impact of inbreeding on growth and agronomic traits (4). Nevertheless, further genotyping of yet missing, intermediate cultivars may reveal a higher connectivity and thus higher inbreeding in the overall pedigree.

*Crosses between cultivars from different regions in Europe*: These were especially frequent from the 19^th^ century onwards (e.g., 'Transparente de Croncels' = 'Calville Blanc d'Hiver' x 'Borowitsky', or 'S.Giuseppe' = 'Abbondanza' x 'Rambour Frank' ).

*Choice of progenitors for further crosses in modern breeding programs*: The cross between 'Dutch Mignonne' and 'White Astrachan' has been reproduced at INRA, Angers, France, with the dual goal of selecting improved full-sibs of the scab resistant cultivar 'Dülmener Rosenapfel' and increasing the frequency of favorable alleles from both parents in elite breeding populations.

*Further extension of pedigree studies*

Special attention should be paid to under-represented genepools such as cider apples or South-Eastern European cultivars. For such work, preliminary genotyping of thousands of accessions with SSR markers has proved to be extremely useful to identify redundancies, mislabelling, and to allocate preferred names for retained cultivars (5).

SNP markers generated with the Axiom®_Apple480K array (6) were available in a very large number thus facilitating robust inferences. Considering the SNP array cost and the requested coverage of the genome for detailed pedigree analysis, a lower density genotyping array such as the Illumina 20K array (7) should be sufficient for further studies. Chloroplast markers (8) could also be very useful to infer the female/male parents in each cross as long as sufficient polymorphism is available within the explored genepool.

**References**

1. Leroy A. Dictionnaire de pomologie: contenant l’histoire, la description, la figure des fruits anciens et des fruits modernes les plus généralement connus et cultivés. Paris: Imprimerie Lachèse, Belleuvre et Dolbeau; 1873.

2. Smith M. National Apple Registry of the United Kingdom. London, UK: Ministry of Agriculture, Fisheries and Food; 1971.

3. Lacombe T, Boursiquot J-M, Laucou V, Vecchi-Staraz MD, Péros J-P, This P. Large-scale parentage analysis in an extended set of grapevine cultivars (*Vitis vinifera* L.). Theor Appl Genet. 2013;126(2):401–14.

4. Brown AG. The effect of inbreeding on vigour and length of juvenile period in apples. In: Proceedings of Eucarpia fruit section Symposium V Top fruit breeding. Canterbury, England; 1973. p. 30–9.

5. Urrestarazu J, Denancé C, Ravon E, Guyader A, Guisnel R, Feugey L, et al. Analysis of the genetic diversity and structure across a wide range of germplasm reveals prominent gene flow in apple at the European level. BMC Plant Biol. 2016;16:130.

6. Bianco L, Cestaro A, Linsmith G, Muranty H, Denancé C, Théron A, et al. Development and validation of the Axiom®Apple480K SNP genotyping array. Plant J. 2016;86(1):62–74.

7. Bianco L, Cestaro A, Sargent DJ, Banchi E, Derdak S, Di Guardo M, et al. Development and Validation of a 20K Single Nucleotide Polymorphism (SNP) Whole Genome Genotyping Array for Apple (*Malus* × *domestica* Borkh). PLoS ONE. 2014;9(10):e110377.

8. Volk GM, Henk AD, Richards CM, Bassil N, Postman J. Chloroplast sequence data differentiate Maleae, and specifically *Pyrus*, species in the USDA-ARS National Plant Germplasm System. Genet Resour Crop Evol. 2019;66(1):5–15.
